# NEDD4 family ubiquitin ligase AIP4 interacts with Alix to enable HBV naked capsid egress in an Alix ubiquitination-independent manner

**DOI:** 10.1371/journal.ppat.1012485

**Published:** 2024-09-11

**Authors:** Sheng Shen, Dawei Cai, Hongyan Liang, Ge Zeng, Wendong Liu, Ran Yan, Xiaoyang Yu, Hu Zhang, Shi Liu, Wanying Li, Rui Deng, Xingyu Lu, Yuanjie Liu, Jian Sun, Haitao Guo

**Affiliations:** 1 Department of Infectious Diseases, State Key Laboratory of Organ Failure Research, Key Laboratory of Infectious Diseases Research in South China, Ministry of Education, Guangdong Provincial Key Laboratory of Viral Hepatitis Research, Nanfang Hospital, Southern Medical University, Guangzhou, China; 2 Department of Microbiology and Molecular Genetics; Cancer Virology Program, UPMC Hillman Cancer Center, University of Pittsburgh School of Medicine, Pittsburgh, Pennsylvania, United States of America; 3 Department of Microbiology and Immunology, Indiana University School of Medicine, Indianapolis, Indiana, United States of America; Pennsylvania State University College of Medicine: Penn State College of Medicine, UNITED STATES OF AMERICA

## Abstract

Hepatitis B virus (HBV) exploits the endosomal sorting complexes required for transport (ESCRT)/multivesicular body (MVB) pathway for virion budding. In addition to enveloped virions, HBV-replicating cells nonlytically release non-enveloped (naked) capsids independent of the integral ESCRT machinery, but the exact secretory mechanism remains elusive. Here, we provide more detailed information about the existence and characteristics of naked capsid, as well as the viral and host regulations of naked capsid egress. HBV capsid/core protein has two highly conserved Lysine residues (K7/K96) that potentially undergo various types of posttranslational modifications for subsequent biological events. Mutagenesis study revealed that the K96 residue is critical for naked capsid egress, and the intracellular egress-competent capsids are associated with ubiquitinated host proteins. Consistent with a previous report, the ESCRT-III-binding protein Alix and its Bro1 domain are required for naked capsid secretion through binding to intracellular capsid, and we further found that the ubiquitinated Alix binds to wild type capsid but not K96R mutant. Moreover, screening of NEDD4 E3 ubiquitin ligase family members revealed that AIP4 stimulates the release of naked capsid, which relies on AIP4 protein integrity and E3 ligase activity. We further demonstrated that AIP4 interacts with Alix and promotes its ubiquitination, and AIP4 is essential for Alix-mediated naked capsid secretion. However, the Bro1 domain of Alix is non-ubiquitinated, indicating that Alix ubiquitination is not absolutely required for AIP4-induced naked capsid secretion. Taken together, our study sheds new light on the mechanism of HBV naked capsid egress in viral life cycle.

## Author summary

HBV-infected cells can produce various viral particles with different sizes and morphologies. Virions are complete particles with both nucleocapsids and surface antigen (HBsAg) envelope, which exploits the endosomal sorting complexes required for transport (ESCRT)/multivesicular body (MVB) pathway for budding. In addition to virion, HBV-infected cells also secrete non-enveloped (or naked) capsid particles. It remains unclear how HBV naked capsid is released from the infected cells. In this study, we further characterized the naked capsid and identified the Lysine 96 of HBV core protein as an important contact point for cellular protein Alix to mediate naked capsid secretion. We also determined NEDD4 E3 ubiquitin ligase family member AIP4 stimulates the release of naked capsid, regardless of their genome contents. AIP4 interacts with Alix and promotes its ubiquitination. However, the AIP4 protein *per se* rather than its E3 ubiquitin ligase activity is sufficient for Alix-induced naked capsid secretion. In summary, these findings provide new insights into a better understanding of HBV naked capsid egress.

## Introduction

Hepatitis B virus (HBV) infection is a leading cause of chronic liver diseases and remains a substantial global public health burden affecting over 257 million people [1]. HBV is a hepatotropic enveloped DNA virus containing a 3.2-kb partially double-stranded circular DNA (rcDNA) genome, which belongs to the *Hepadnaviridae* family [2,3]. Upon infection of hepatocytes by exploiting the hepatocyte-specific receptor sodium taurocholate cotransporting polypeptide (NTCP), the viral rcDNA genome is transported into the nucleus to form the covalently closed circular DNA (cccDNA), which served as the template for transcription of all viral RNAs, including the 3.5-kb precore (pc) and pregenomic (pg) RNA, 2.4/2.1-kb surface mRNAs, and 0.7-kb X mRNA [4]. The pgRNA is not only the template for HBV reverse transcription but also the template for translating viral core and polymerase (pol) proteins. The binding of pol to the epsilon (ε) at the 5’ terminal region of pgRNA recruits core proteins to encapsidate the pgRNA/pol complex into nucleocapsid, inside of which the pol reverse transcribes pgRNA into the single-stranded DNA (ssDNA) and then the rcDNA [5,6]. The newly synthesized rcDNA-containing nucleocapsid is either enveloped by surface proteins and secreted as the complete virion or recycles the progeny rcDNA into cell nucleus to replenish the cccDNA pool [2,7]. In addition to the DNA-containing virions, other secreted forms of non-infectious HBV particles have been found *in vivo* and/or *in vitro*, including the subviral particles (HBsAg), the empty virion without viral nucleic acids, the RNA-containing virions, and the non-enveloped (naked) capsids [7–9].

The endosomal sorting complex required for transport (ESCRT) mediates the scission of membrane necks formed when membrane-bound vesicles bud in a direction away from the cytoplasm [10]. The ESCRT pathway functions in a variety of cellular processes including multivesicular body (MVB) formation, budding of microvesicles, exosome formation, cytokinesis and an expanding list of other cellular processes [11]. This ESCRT machinery consists of five protein complexes known as ESCRT-0, -I, -II, -III, and VPS4 ATPase complex [12]. A wide range of viruses use the ESCRT pathway to facilitate release of new virions, which has been well-characterized with the human immunodeficiency virus (HIV) [13]. Like many other enveloped viruses, HBV egress also requires the recruitment of the ESCRT/MVB pathway, which is corroborated by the findings that inactivation of ESCRT-II/III or VPS4 potently inhibited HBV DNA-containing virions budding [14,15], and knockdown of ESCRT-I component TSG101 reduced the number of HBV virion particles in MVB [16], although another study reported that RNA interference of ESCRT-I subunits TSG101 and VPS28 did not block, but rather stimulate virus release [15]. In addition to the infectious virions, the filamentous HBsAg particles and the RNA-containing virions are also released via the ESCRT/MVB pathway, whereas the spherical HBsAg particles egress from the cells through the endoplasmic reticulum (ER)-Golgi constitutive secretory pathway [7,17]. In addition, the nonparticulate HBV e antigen (HBeAg), a homologue of core protein, is also secreted via the ER-Golgi secretion pathway [18–20]. However, it remains largely unclear how HBV naked capsids are released from cells. Previous studies have reported that the secretion of naked capsid does not require the ER-Golgi pathway or an integral ESCRT/MVB pathway [21,22], but it involves the ESCRT-III-binding protein Alix and ESCRT-0 component HGS [23,24]. Our recent study has shown that the inhibition of VPS4 in the ESCRT/MVB secretory pathway did not affect the egress of naked capsid in cell cultures [25]. Therefore, the detailed secretory mechanism/pathway of naked capsid awaits further investigation.

The 21 KDa HBV core (HBc) protein plays key structural roles in multiple steps of HBV life cycle, including capsid assembly, pgRNA packaging, reverse transcription, virion secretion, and cccDNA formation [26–29]. There are two conserved Lysine residues, K7 and K96, located on the surface of HBc dimer and capsid [21,30,31]. Lysine residues potentially undergo multiple types of reversible post-translational modifications (PTMs), such as ubiquitination, SUMOylation, NEDDylation, acetylation, and methylation, which are crucial for regulating protein stability, enzymatic activity, signaling transduction, etc [32]. However, the biological role of the HBc Lysine residues and their possible PTMs in HBV lifecycle remain vague. While the direct evidence for K7 ubiquitination of overexpressed HBc has been obtained by mass spectrometry [33,34], the potential ubiquitination of K96 and SUMOylation of K7/96 were mainly suggested by forced expression of ubiquitin (Ub) and SUMO modifiers in mutagenesis studies [16,35]. Previous studies have been focused on the effects of HBc K7/96 mutations on nucleocapsid formation and virion secretion. It appeared that the substitution of K7 and/or K96 with different residues exhibited no or less alteration of capsid assembly, pgRNA encapsidation, reverse transcription, or cccDNA formation [16,31,36–39]. While K7 mutations did not affect virion secretion, substitutions of K96 with alanine (A) or Arginine (R) blocked virion production in several studies [31,38,39]; and it has been reported that K96 can be ubiquitinated by NEDD4 and subsequently interacts with γ2-adaptin or TSG101 for MVB-mediated virion secretion [16,37]. However, another study demonstrated that either K7R or K96R mutant had no influence on HBV virion release from transfected cells [36]. Despite the reported controversial role of HBc K96 in virion secretion, thus far, to the best of our knowledge, no study has been focused on the role of HBc K7/96 residues and their PTMs, if any, in the egress of naked capsid in cell cultures.

In this study, we set out to systematically study the biogenesis and molecular characteristics of HBV naked capsids, as well as decipher the relationships among HBc Lysine residues, their potential ubiquitination, HBV replication, and naked capsid egress.

## Results

### Characterization of extracellular hepadnaviral naked capsids

**Fig 1:**
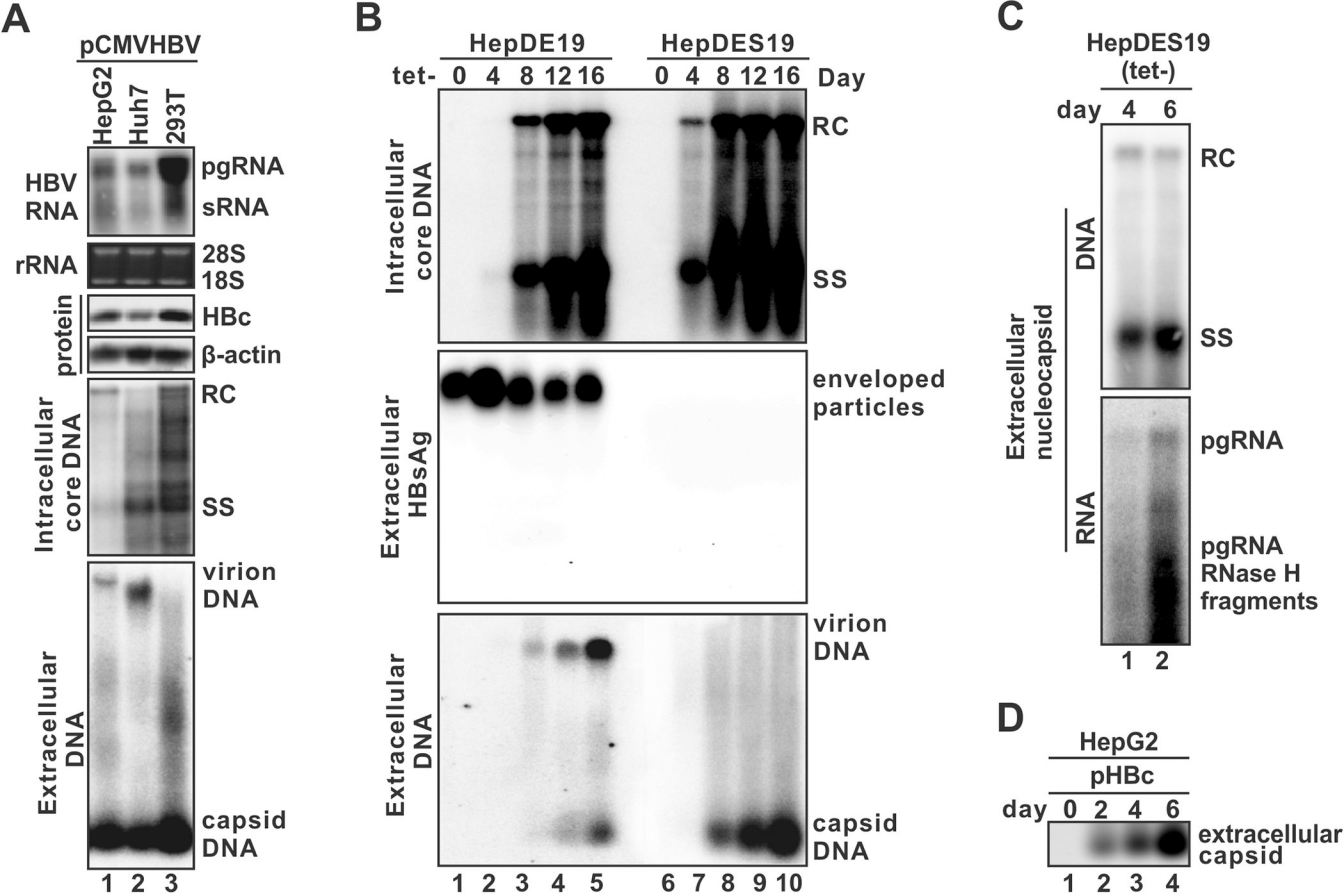
The existence and genome content of HBV naked capsid in cell cultures. (A) Naked capsids are present in the supernatant of HBV transiently transfected cells. Plasmid pCMVHBV was transfected into HepG2, Huh7, and 293T cells for 5 days. The intracellular viral total RNA, core protein, core DNA and extracellular viral particles were analyzed by Northern bot, Western blot, Southern blot, and native particle gel assay, respectively. The 28S and 18S ribosomal RNA (rRNA) and β-actin served as loading control for total RNA and protein, respectively. HBV pgRNA and surface mRNA (sRNA) were hybridized with a plus (+) strand-specific full-length HBV riboprobe. Intracellular and extracellular HBV core (capsid) DNA was probed with a minus (-) strand-specific full-length HBV riboprobe. The positions of relaxed circular (RC) and single-stranded (SS) DNAs are marked on Southern blot, and HBV DNA-containing virions and naked capsids are indicated on particle gel. (B) The presence of naked capsids in the supernatant of HBV stably transfected cells. HBV replication was induced in HepDE19 and HepDES19 cells with tet-free medium for the indicated time duration. The cytoplasmic HBV capsid DNA intermediates were analyzed by Southern blot. HBV particles in culture fluids were analyzed by particle gel. Enveloped HBV particles, including virions and subviral particles (HBsAg), were revealed by immunoblot with HBsAb. HBV virion DNA and naked capsid DNA were detected by (-) strand-specific riboprobe. (C) HBV capsid-associated DNA and RNA were extracted from the supernatant of induced HepDES19 cells and subjected to Southern and Northern blotting, respectively. The smear signal below the pgRNA band represents the pgRNA fragments degraded by the viral RNase H during reverse transcription. (D) HBV genome-free (empty) naked capsid is secretion-competent. HepG2 cells were transfected with pHBc for the indicated time duration, and the naked capsid in the supernatant was detected by particle gel immunoblot with HBcAb.

Previous studies have shown that the supernatant of HBV-replicating hepatoma cell lines contains naked capsid in addition to enveloped virions and envelope-only subviral particles (SVP/HBsAg) [22–24,40–47]. However, less attention has been paid to the naked capsid as HBV is an enveloped virus *per se* and the naked capsid is considered non-infectious. Nonetheless, it remains interesting to understand the biogenesis, characteristics, and potential biological functions of naked capsid in HBV life cycle. To this end, we set out to systematically determine the existence and characteristics of naked capsid from *in vitro* and *in vivo* samples. Firstly, we examined the naked capsids from different cell lines transiently transfected with HBV producing plasmid pCMVHBV. Owing to different cell origins and transfection efficiencies, HBV reproduction levels including total viral RNA, cytoplasmic core DNA, and secreted DNA-containing virion were variable among hepatoma cell lines HepG2, Huh7, and non-hepatic 293T cells, but high levels of DNA-containing naked capsids were detected in the supernatants of all three cells by particle gel assay (Fig 1A). Moreover, upon induction of HBV replication in both wild type (wt) HepDE19 and envelope-null HepDES19 stable cell lines, naked capsids were also found to be detected and accumulated in the culture fluid of both cells over time, together with the secreted virions and SVP/HBsAg in HepDE19 cells (Fig 1B). The sucrose density gradient fractionation plus capsid/particle gel analyses of cell lysate and supernatant samples of induced HepDE19 cells further confirmed the existence of extracellular naked capsid (S1 Fig). Hence, naked capsids are ubiquitously present in the supernatant of HBV replicating cells.

Since HepDES19 cell line does not express HBV envelope proteins, it only releases naked capsids upon induction of virus replication (Fig 1B) [40], which also indicates that the observed naked capsid particles in the supernatant is not released from the secreted virions but rather from a specific secretion event. We thus used this cell line to further characterize the nucleic acid contents of naked capsids. HBV particles were pelleted down from the supernatant of induced HepDES19 cells, viral capsid DNA and RNA were then extracted and subjected to Northern and Southern blots. We found that pgRNA-containing capsids, ssDNA-containing capsids, and rcDNA-containing capsids were all detected in a mixture of naked capsids (Fig 1C). Furthermore, we transfected HepG2 cells with a plasmid only expressing HBc protein and found that the “empty” capsids without viral RNA or DNA can be released into cell supernatant as well (Fig 1D). The above results imply that all immature and mature HBV capsids can be released from cells as naked capsid, regardless of HBV nucleic acid content within the capsids or viral replication steps.

Next, we assessed whether HBV naked capsids can be detected from *in vivo* HBV experimental systems. The pooled serum samples previously collected from HBV transgenic mice and virally infected uPA/SCID mice with humanized liver were analyzed by particle gel assay. As shown in S2A Fig, HBV virion particles were detected in both murine serum samples, however, naked capsids were only observed in sera of HBV-infected humanized mice but not the HBV transgenic mice. Considering that antibodies against HBc (HBcAb) are rapidly and robustly induced and persist during the cause of HBV infection [48], we reasoned that the lack of HBcAb in the immunodeficient humanized mice accounts for the presence of circulating naked capsid. In line with this notion, HBcAb was only detected in the immunocompetent HBV transgenic mice sera but not in HBV-infected humanized mice sera or the control supernatant from induced HepDE19 cells (S2B Fig), which is consistent with previous reports that the highly immunogenic HBV capsid could be cleared in circulation by HBcAb in immunocompetent mice [49–52].

We further examined the potential existence of naked capsid in the context of duck hepatitis B virus (DHBV) replication or infection. In DHBV-positive duck serum, only virions were detected by particle gel assay (S3 Fig, lane 1), the absence of naked capsid is consistent with the result from HBV transgenic mice serum (S2 Fig). However, naked capsids were detected together with virions in the supernatant samples of p2DHBV-trasnfected LMH chicken hepatoma cells and DHBV stably transfected LMH-D2 cells (S3 Fig, lanes 2 and 4). As expected, the envelope-null DHBV inducible cell line dstet5 only released naked capsid to the supernatant (S3 Fig, lane 3). Collectively, the results demonstrate that the release of naked capsids into cell culture fluid is a common feature of hepadnaviruses.

### HBc K96R mutation abolishes naked capsid release without affecting intracellular viral replication

**Fig 2:**
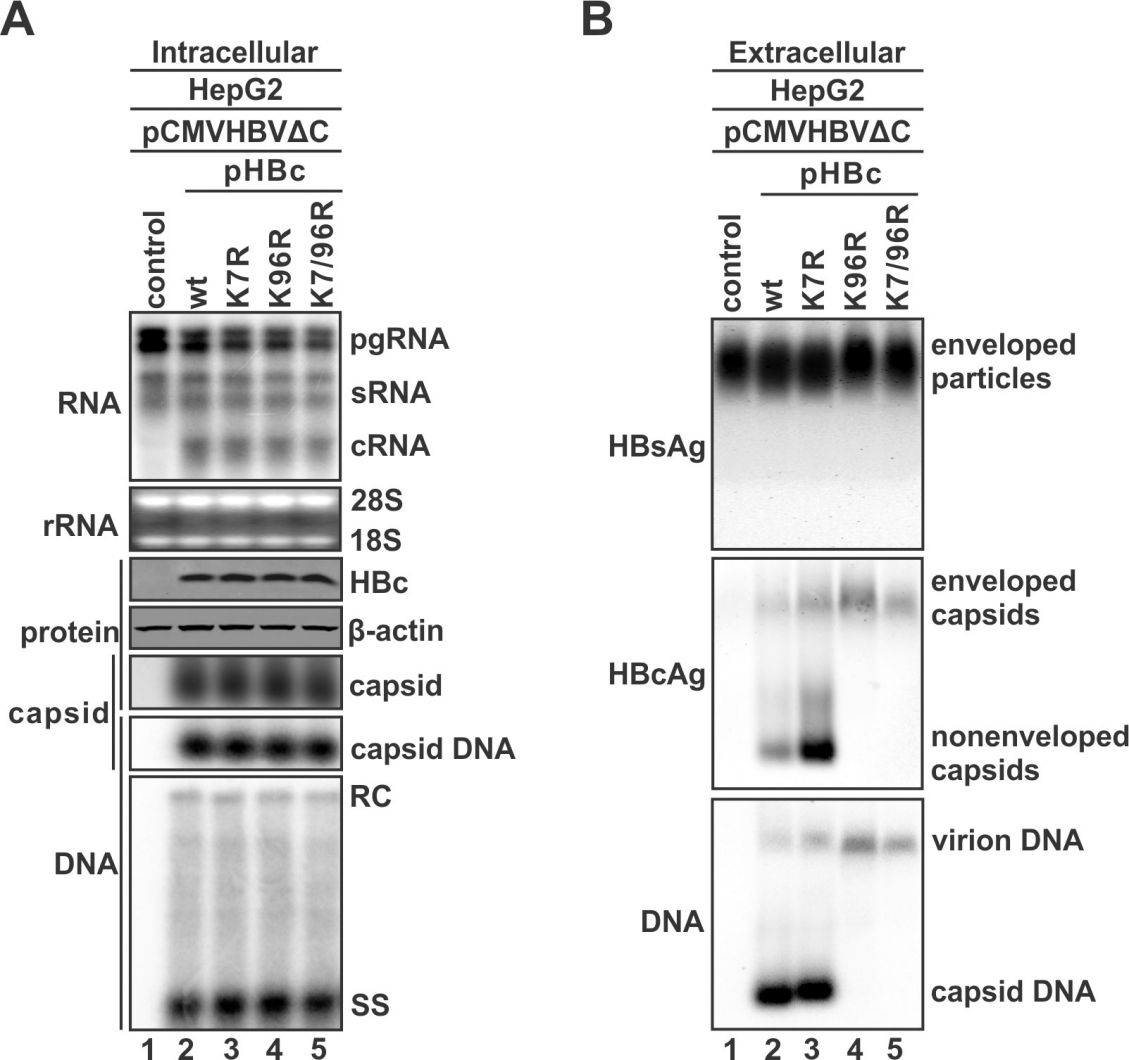
The effects of HBc K7R and K96R mutations on HBV replication and viral particle secretion. The HBc-null HBV plasmid pCMVHBVΔC and control vector or plasmid expressing wt HBc, K7R, K96R, or K7R/K96R mutants were co-transfected into HepG2 cells for 5 days. (A) Cells were harvested and the intracellular HBV total RNA, HBc protein, capsids and total DNA content, and capsid DNA intermediates were analyzed by Northern bot (upper panel), Western blot (upper middle panels), capsid gel (lower middle panels), and Southern blot (lower panel), respectively. HBV pgRNA, sRNA, and wt and mutant HBc-coding mRNA (cRNA) were hybridized with a plus (+) strand-specific full-length HBV riboprobe. Cytoplasmic HBV capsids and the encapsidated HBV DNA were detected by capsid gel immunoblot and hybridization, respectively. Cytoplasmic HBV capsid DNA intermediates on Southern blot were probed with a minus (-) strand-specific full-length HBV riboprobe. (B) HBV particles in culture fluids were detected by particle gel assay. The enveloped viral particles, including virions and subviral particles (HBsAg), were probed by HBsAb (upper panel), virions and naked capsids were probed by HBcAb (middle panel), virion DNA and naked capsid DNA were detected by HBV (-) strand-specific riboprobe (lower panel). The positions of HBV enveloped particles and naked capsids are indicated.

The two highly conserved Lysine residues, K7 and K96, located on the surface of HBV core protein/capsid of all known orthohepadnaviruses, have been studied for their potential roles in multiple steps of HBV replication cycle, including capsid assembly, DNA replication, cccDNA formation, and virion morphogenesis, through site-specific and combinational mutagenesis [16,31,36–39]. Among those studies, while K7 has been shown to be dispensable in the above HBV reproduction events, controversial results were obtained regarding the involvement of K96 in the ESCRT/MVB-mediated secretion of HBV virion [16,31,36,37,39,53]. However, the possible effect of K7 and K96 on naked capsid release has not been rigorously studied. To address this research question, we substituted the individual Lysine or both to the conservative Arginine (R), giving rise to the following three HBc mutants: K7R, K96R, and K7R/K96R. We then co-transfected these mutants with the HBc-null HBV plasmid pCMVHBVΔC into human hepatoma cells to analyze the potential roles of K7/96 in different stages of HBV life cycle. As shown in Fig 2A, while pCMVHBVΔC alone produced viral pgRNA and surface mRNAs without supporting capsid formation and DNA replication in HepG2 cells due to the absence of HBc expression, trans-complementation with wt HBc or each above K-to-R mutant resulted in comparable levels of viral RNA transcription, HBc protein expression, cytoplasmic capsid formation, and core DNA synthesis. These results are consistent with a recent study [53]. Next, we examined the potential effect of these mutations on HBV particle secretion by using the native particle gel assay. As shown in Fig 2B, comparable levels of enveloped HBV particles (virions and SVPs) were observed in the supernatant of all transfected cells by HBsAg immunoblotting (top panel); when the membrane was probed with HBc-specific antibody and (-) strand-specific HBV riboprobe, respectively, signals were detected in virions and naked capsids derived from the wt HBc and K7R mutant, whereas the K96R and K7/96R mutants completely lost naked capsid signal without reducing the virion signals (bottom panels).

To exclude the possibility that any step(s) or product(s) of HBV replication may influence naked capsid release, wt HBc or each K-to-R mutant alone was transfected into HepG2 cells for assessment of naked capsid production. The particle gel assay showed consistent results that K96R and K7/96R abolished the release of empty naked capsid into supernatant without reducing the intracellular capsid formation (S4 Fig). Thus, K96R is the determining mutation for this defective naked capsid-release phenotype. To further confirm the inhibition of naked capsid release by K96R mutation, we conducted transmission electron microscopy (TEM) analysis. As shown in S5 Fig, while large quantity of intracellular capsids was observed from both wt HBc- and K96R-transfected HepG2 cells, extracellular naked capsids were only found from supernatant of wt HBc- but not K96R-transfected cells under TEM.

To assess one possibility that the release of naked capsid from cells is due to cell death or alteration of plasma membrane integrity, we performed the Homogeneous Membrane Integrity Assay by measuring the release of lactate dehydrogenase (LDH) from cells upon plasma membrane damage. The results demonstrated that the cell membrane integrity remained unchanged in cells transfected with either wt HBc or each K-to-R mutants compared to control vector (S6 Fig), indicating that the egress of naked capsid and the K96R phenotype do not involve cytotoxicity or cell membrane damage issues.

### The K96R mutation of precore protein does not affect HBeAg secretion

HBV precore/core (pC/C) ORF contains two in-frame start codons that encode precore and core protein, respectively. The pC protein bears a 29 aa N-terminal extension than HBc, and it is the precursor for the secreted hepatitis B e antigen (HBeAg) (S7A Fig) [3]. We thus examined whether the K96R mutation of pC protein could affect HBeAg secretion. The results showed that K96R did not affect the expression of intracellular pC (p22 with signal peptide being cleaved); while wt pC and K96R mutant did not form capsid as anticipated, it appeared that the HBeAg secretion was not affected by K96R mutation (S7B and S7C Fig). Since HBeAg secretion takes the ER/Golgi secretory pathway as HBsAg does [7], the differential effects of K96R on naked capsid and HBeAg secretion indicate that the latter utilizes a distinct secretory mechanism.

### K96 is required for the interaction between capsid and ubiquitin

**Fig 3:**
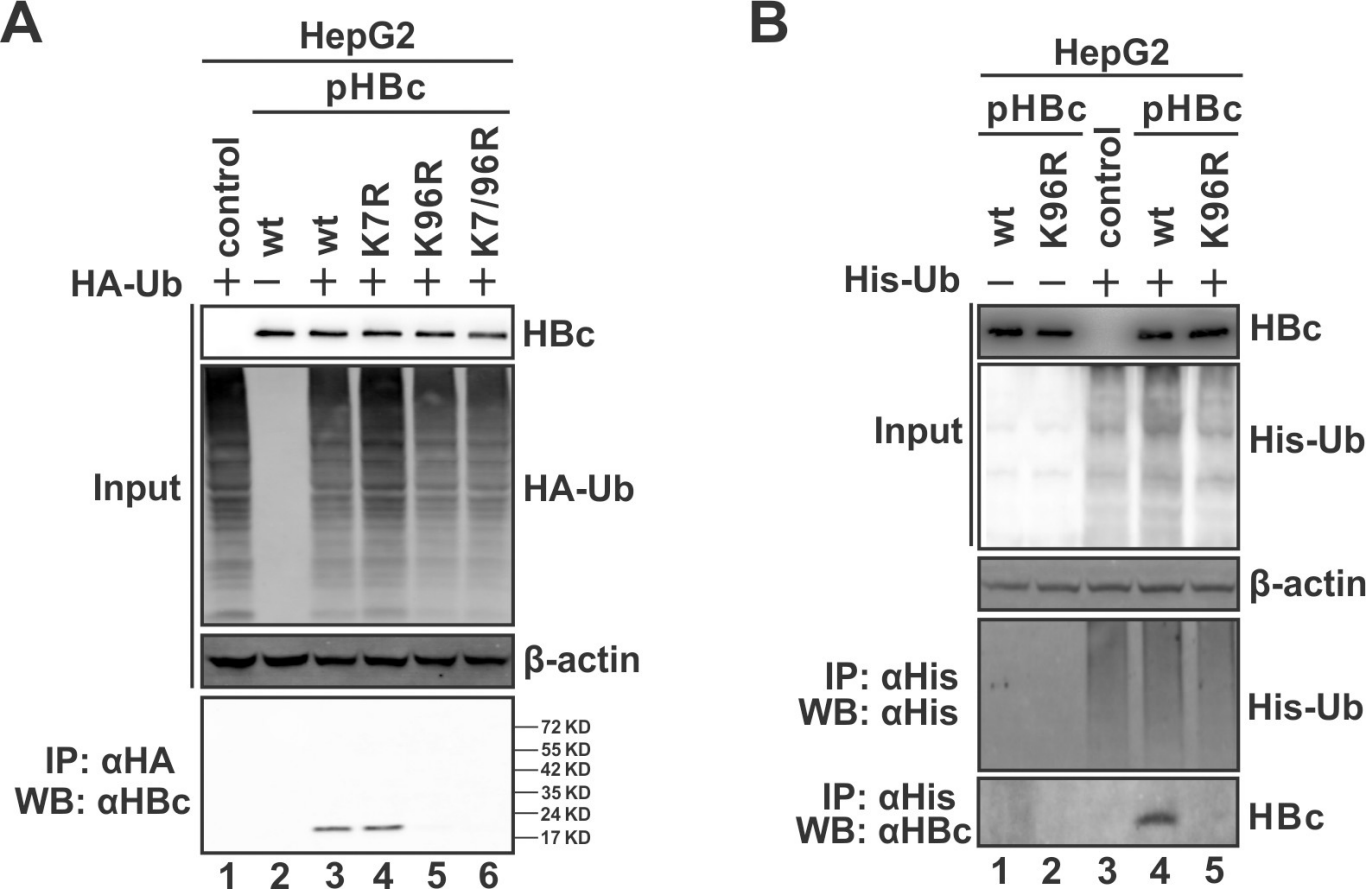
K96 is required for the interaction between HBc and ubiquitin. (A) HepG2 cells were co-transfected with HA-ubiquitin (HA-Ub) and wt or mutant HBc as indicated for 3 days. Transfection of HA-Ub or wt HBc alone served as control. Protein expressions were analyzed by Western blot. The smears on the HA-Ub blot indicate the ubiquitinated proteins. The cytoplasmic lysate was subjected to immunoprecipitation of HA-Ub and the co-immunoprecipitated HBc was detected by Western blot. (B) HepG2 cells were transfected by wt HBc or K96R with or without His-Ub for 3 days. His-Ub transfection alone served as control. The expression of transfected proteins was detected by Western blot, β-actin served as a loading control. Immunoprecipitation of His-Ub was performed, followed by the detection of His-Ub ubiquitinated proteins and co-immunoprecipitated HBc by Western blot.

Considering that K96 has been implicated to interact with ESCRT components in a ubiquitination-dependent manner for virion secretion in previous studies [16,37]. The observed K96R phenotype of naked capsid egress raised a question on whether K96 could be ubiquitinated on HBc or capsid. To address this, we co-transfected HepG2 cells with wt HBc or K-to-R mutants and the HA-ubiquitin (HA-Ub) or His-ubiquitin (His-Ub), and the potential physical association between HBc and ubiquitin was examined by co-immunoprecipitation (co-IP). As shown in Fig 3, the naked capsid secretion-competent wt HBc and K7R mutant, but not the secretion-defective K96R mutant or K7R/K96R double-mutant, were indeed pulled down with the ectopically expressed ubiquitin. However, it appeared that the immunoprecipitated wt HBc or K7R mutant was not ubiquitinated, at least the polyubiquitinated HBc bands were not observed under our experimental conditions. The results indicate that K96 is required for the association between ubiquitin and the secretion-competent HBc.

### Secretion-competent HBc is not ubiquitinated but associated with ubiquitinated host protein

**Fig 4:**
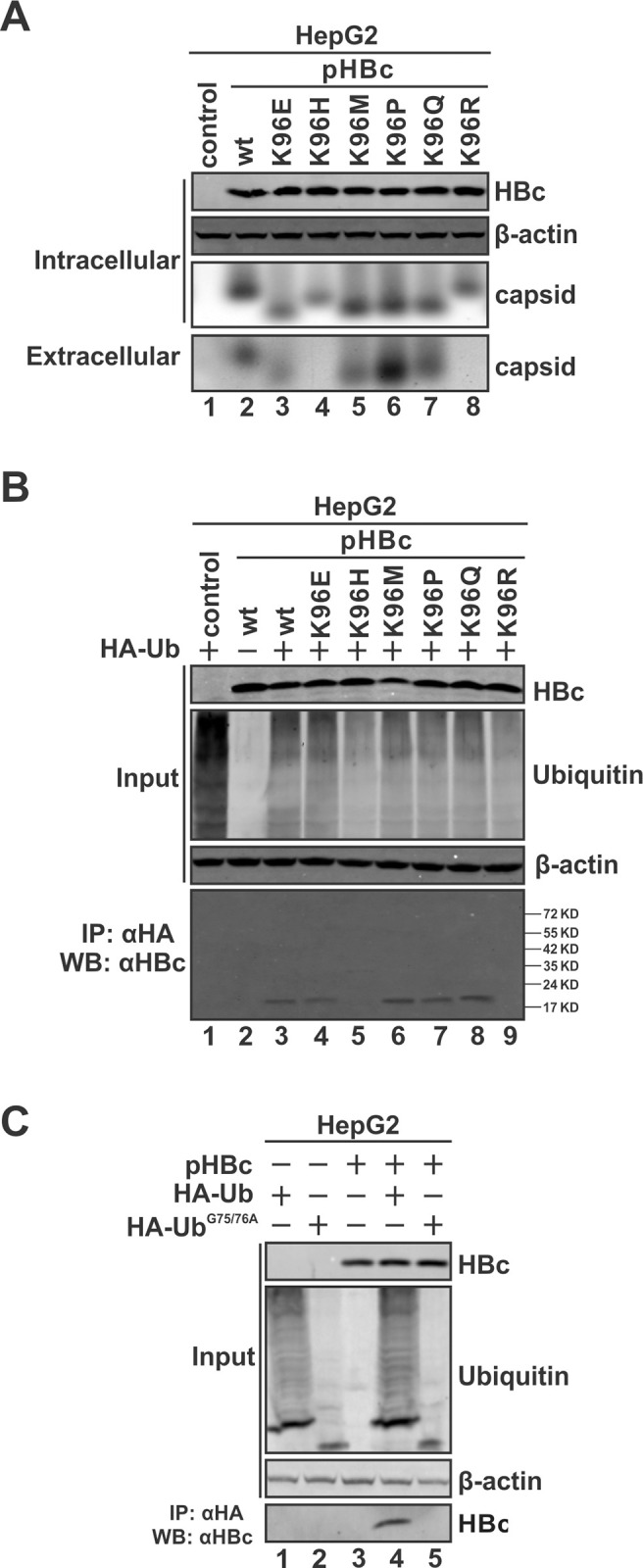
Secretion-competent HBc/capsid is associated with ubiquitin via binding of ubiquitinated host protein(s). (A) HBc K96 mutagenesis analysis. HepG2 cells were transfected with wt HBc or each indicated K96 mutant (K96E, K96H, K96M, K96P, K96Q and K96R) for 3 days. The expression of wt and mutant HBc was detected by Western blot, intracellular and extracellular capsids were analyzed by particle gel immunoblot using HBcAb. (B) HepG2 cells were co-transfected HA-Ub plus wt HBc or each indicated K96 mutant for 3 days. HA-Ub or wt HBc alone served as control. Intracellular expression of wt and mutant HBc, total HA-Ub ubiquitinated proteins, and β-actin was detected by Western blot. HA-Ub immunoprecipitation was performed and the co-immunoprecipitated HBc proteins were detected by Western blot. (C) HepG2 cells were transfected with pHBc, HA-Ub, and Ub conjugation-defective mutant HA-Ub^G75/76A^ in different combinations as indicated. 3 days post-transfection, HBc and total HA-Ub proteins were detected by Western blot, β-actin served as a loading control. HBc co-immunoprecipitated with HA-Ub was detected by Western blot.

Next, we mutated HBc K96 to other representative amino acids, including the negatively-charged polar Glutamic acid (E), aromatic and positively-charged polar Histidine (H), generic hydrophobic Methionine (M), aliphatic and hydrophobic Proline (P), and charge-neutral polar Glutamine (Q), to sort out the common properties of amino acid (aa) position 96 of HBc that determine naked capsid secretion. As shown in Fig 4A, all these mutants were expressed at comparable levels and supported capsid assembly like wt HBc and K96R mutant. Notably, capsids formed by K96E, K96H, K96M, K96P and K96Q migrated faster than wt and K96R in the native capsid gel, which might be due to the alteration of charge on the capsid surface and/or capsid size/mass caused by Lysine substitution. Furthermore, while K96E, K96M, K96P and K96Q capsids remained secretion-competent, only the K96H mutant exhibited secretion-defective phenotype as K96R did.

We then examined the association between these additional K96 mutants and ubiquitin by co-IP. Intriguingly and consistently, the results demonstrated that the wt capsid and secretion-competent K96 mutants exhibited association with ubiquitin, only the secretion-defective mutants K96H and K96R failed to interact with ubiquitin (Fig 4B). Considering that the non-Lysine residues (except for Histidine and Arginine) at aa 96 are able to support naked capsid secretion, thus, K96 ubiquitination, if any, is not absolutely required for naked capsid secretion. Additionally, the observed association of ubiquitin with secretion-competent K96 mutant naked capsid indicates that it is not a ubiquitination of HBc.

Then, there are two possibilities for the capsid-associated ubiquitin: 1) a non-covalent affinity binding of ubiquitin to capsid; 2) a ubiquitinated host protein(s) interacting with capsid. It is known that the carboxyl group of the C-terminal Glycine residue of ubiquitin (G76) serves as the moiety conjugated to substrate Lysine residues [54]. We thus co-transfected the wt HA-Ub or the conjugation-deficient mutant HA-Ub^G75/76A^ with wt HBc into HepG2 cells. The co-IP assay revealed that the conjugation-deficient ubiquitin failed to be coimmunoprecipitated with HBc (Fig 4C). Hence, the interaction between HBc/capsid and ubiquitin is likely indirect and certain ubiquitinated host protein(s) is involved in naked capsid secretion.

### Ubiquitinated Alix is associated with egress-competent HBV capsids

**Fig 5:**
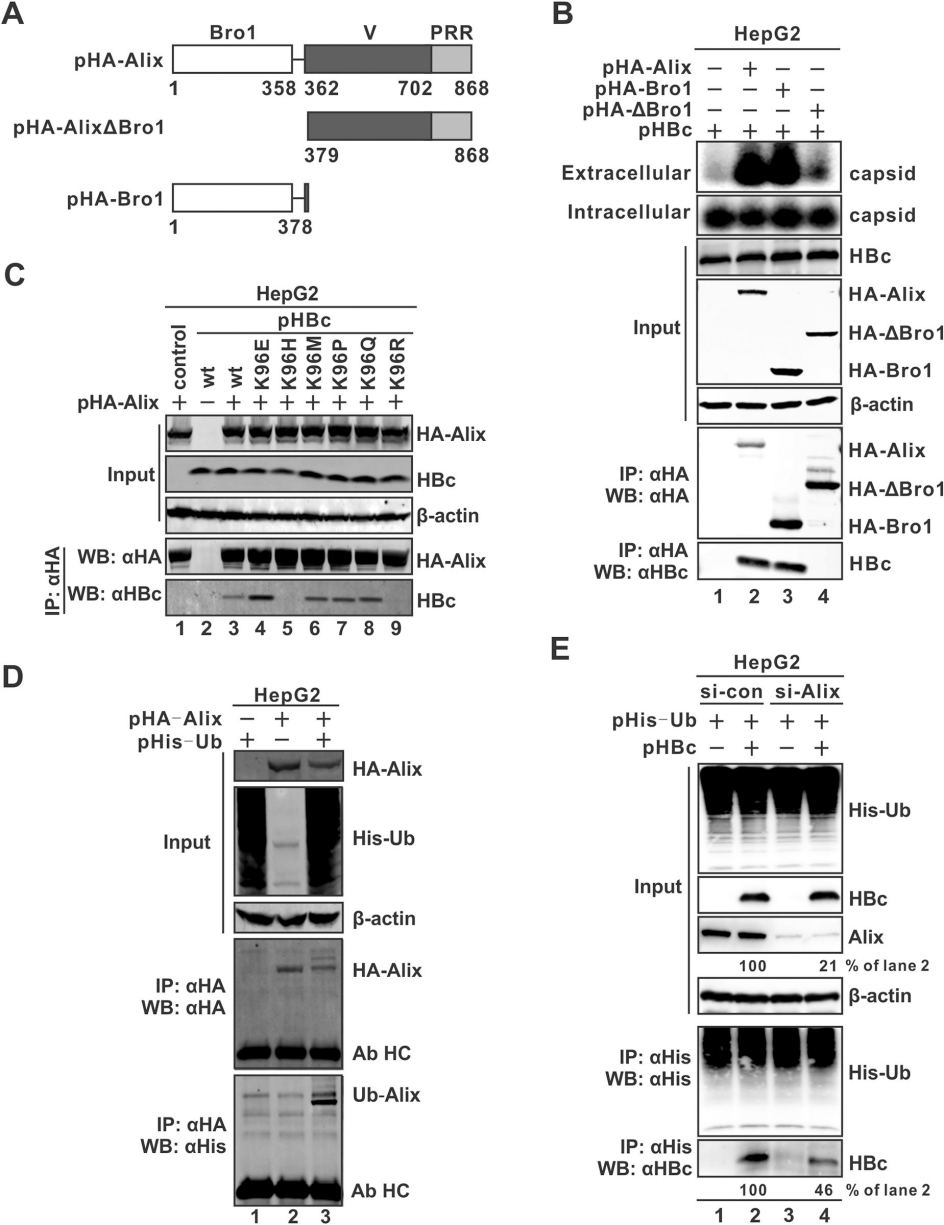
Secretion-competent HBc/capsid is associated with ubiquitinated Alix. (A) Schematic representation of wt Alix and domain-deletion mutants. (B) Alix interacts with HBc and promotes naked capsid secretion via the Bro1 domain. The HA-tagged full-length Alix, Bro1 only, or ΔBro1 was co-transfected with wt HBc into HepG2 cells for 3 days. Intracellular and extracellular capsids were detected by particle gel immunoblot. The expression of transfected proteins was detected by Western blot, β-actin served as a loading control. HA-tagged Alix and mutants were immunoprecipitated and the co-immunoprecipitated HBc was detected by Western blot. (C) Alix interacts with egress-competent HBc. HA-Alix was transfected into HepG2 cells plus wt HBc or each indicated K96 mutants for 3 days. Protein expression was detected by Western blot. HA-Alix co-immunoprecipitation was performed and the immunoprecipitated HA-Alix and HBc were detected by Western blot. (D) Ubiquitination of Alix. HepG2 cells were transfected with HA-Alix or His-Ub or in combination for 3 days. The expression of HA-Alix, total His-Ub-ubiquitinated proteins, and β-actin loading control was detected by Western blot. Co-immunoprecipitation of HA-Alix was performed, and the immunoprecipitated HA-Alix and ubiquitinated Alix (Ub-Alix) were detected by Western blot. The co-detected antibody heavy chain (Ab HC) was indicated. (E) Alix is the major HBc-interacting ubiquitinated protein. HepG2 cells were treated with control siRNA (si-con) or Alix siRNA (si-Alix) for 24 h, followed by transfection with His-Ub alone or together with pHBc for 3 more days. The His-Ub-tagged proteins, HBc, and endogenous Alix were detected by Western blot, β-actin served as a loading control. Co-immunoprecipitation of His-Ub was performed, and the immunoprecipitated His-Ub-tagged proteins and HBc were detected by Western blot.

Previous studies reported that the ESCRT accessory protein Alix (ALG2-interacting protein X) and the ESCRT-0 component HGS regulate the secretion of HBV naked capsid [23,24]. Since HGS appears to promote naked capsid release in a ubiquitin-independent manner [24], we thus investigated whether Alix was the ubiquitinated protein interacting with egress-competent HBV capsid. Human Alix consists of three regions, and the N-terminal Bro1 domain has been reported to be required for Alix to interact with HBc and promote naked capsid egress (Fig 5A) [23]. Consistent with this, our results confirmed that wt Alix and the Bro1-domain alone, but not the Bro1-deletion mutant ΔBro1, significantly facilitated naked capsid egress without affecting the levels of intracellular HBc and capsid (Fig 5B, top panels). Moreover, wt Alix and Bro1, but not ΔBro1, demonstrated the interaction with HBc in the reciprocal co-IP assay (Fig 5B, lower panels). In addition, siRNA knock-down of the endogenous Alix markedly reduced naked capsid secretion (S8 Fig). Next, we examined the association between wt HBc and K96 mutants and Alix by co-IP. The results showed that the secretion-competent wt HBc and K96 mutants, but not K96H or K96R, interact with Alix (Fig 5C), suggesting that HBc K96 is a key residue for Alix-mediated naked capsid egress.

The observed association of secretion-competent HBc and mutants with Alix is completely consistent with their interactions with ubiquitin (Figs 4B and 5C), which indicates that Alix may be the ubiquitinated protein associated with egress-competent capsid. We thus examined whether Alix can be ubiquitinated by co-transfecting HepG2 cells with Alix and His-Ub, followed by co-IP assay. The result demonstrated that Alix can be poly-ubiquitinated as observed by the multiple bands of immunoprecipitated HA-Alix (Fig 5D, lane 3). The similar result was also obtained in non-hepatic 293T cells (S9 Fig), indicating that Alix ubiquitination does not require hepatocyte-specific factors. Furthermore, the interaction between HBc and ubiquitin was markedly reduced upon knocking down the endogenous Alix by siRNA (Fig 5E). The above results demonstrated that the ubiquitinated Alix is associated with intracellular egress-competent HBV capsid and facilitates the egress of naked capsid.

### NEDD4 family E3 ubiquitin ligase AIP4 promotes HBV naked capsid egress

**Fig 6:**
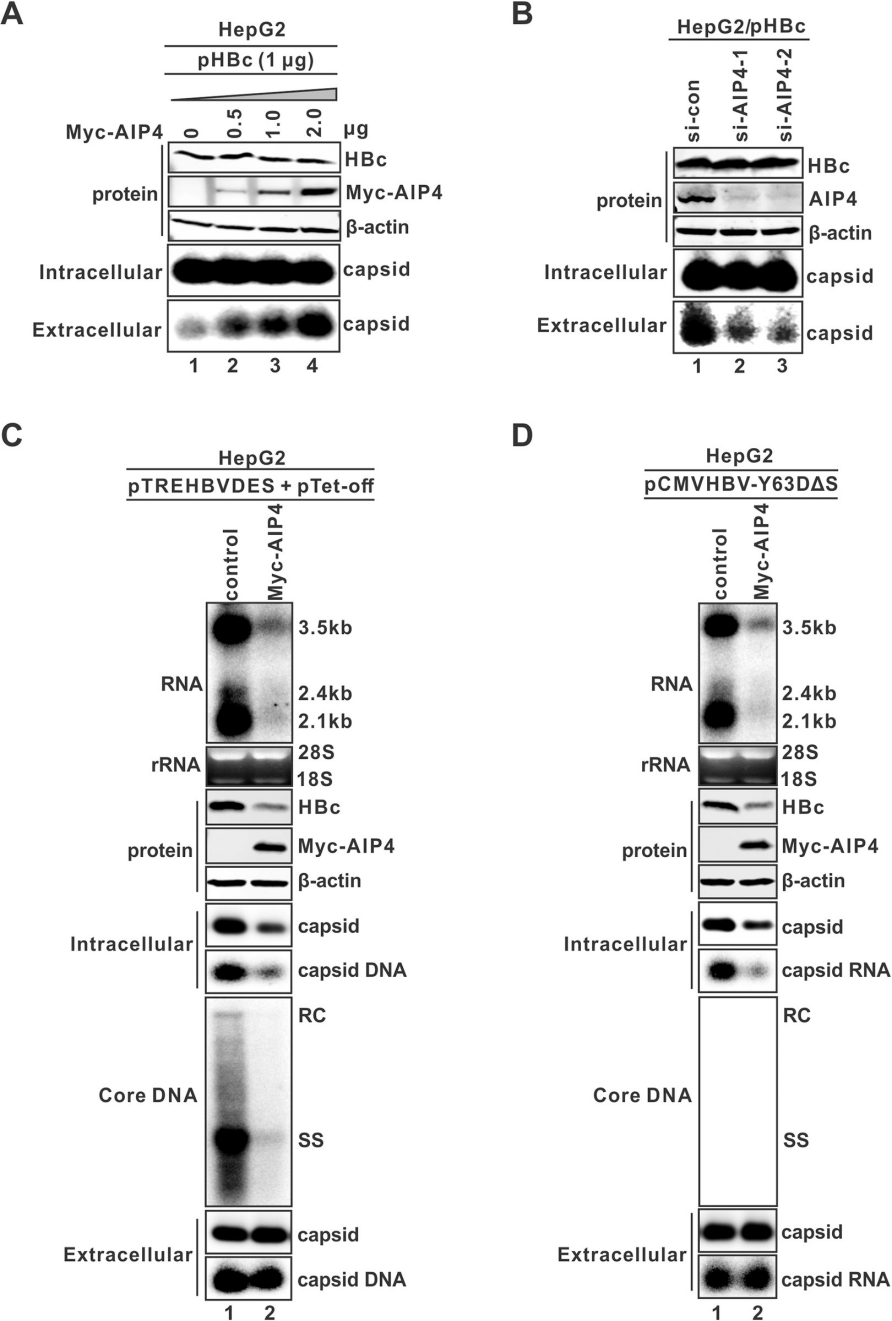
NEDD4 family E3 ubiquitin ligase AIP4 promotes HBV naked capsid egress. (A) AIP4 ectopic expression dose-dependently promotes naked capsids secretion. HepG2 cells were co-transfected with 1.0 μg of HBc and the indicated amounts of Myc-tagged AIP4 (Myc-AIP4). The control vector was supplemented to normalize the total amount of transfected plasmids to 3.0 μg per transfection. Cells were harvested at day 3 post-transfection, and the expression of Myc-AIP4 and HBc was analyzed by Western blot, intracellular and extracellular capsid were detected by particle gel immunoblot. (B) Knockdown of endogenous AIP4 inhibits HBV naked capsid secretion. HepG2 cells were transfected with si-con or AIP4 siRNA (si-AIP4-1 and -2 with different sequence targets) for 24 h, followed by pHBc transfection for 3 more days. HBc expression and knockdown efficiency of endogenous AIP4 were analyzed by Western blot, intracellular and extracellular capsids were detected by particle gel. (C) Overexpression of AIP4 promotes the egress of HBV DNA-containing naked capsid. HepG2 cells were co-transfected with envelope-null pTREHBVDES and pTet-off together with a control vector or Myc-AIP4 for 5 days. The intracellular HBV total RNA, HBc, Myc-AIP4, cytoplasmic HBV capsid and DNA content, and core DNA intermediates were analyzed by Northern bot (upper panel), Western blot (upper middle panels), capsid gel (middle panels), and Southern blot (lower middle panel), respectively. Extracellular naked capsids and viral DNA content were detected by particle gel immunoblot with HBcAb and hybridization with (-) strand-specific HBV riboprobe, respectively. (D) AIP4 promotes the egress of HBV pgRNA-containing naked capsids. HepG2 cells were co-transfected with priming-defective and envelope-null pCMVHBVY63DΔS and control vector or Myc-AIP4 for 3 days. The intracellular HBV total RNA, HBc, Myc-AIP4, cytoplasmic HBV capsid and core DNA intermediates, and extracellular naked capsids were analyzed as above described. The intracellular and extracellular capsid-associated HBV RNA were detected by particle gel hybridization using a (+) strand-specific full-length HBV riboprobe.

The covalent conjugation of ubiquitin to target proteins is accomplished through a stepwise enzymatic reaction cascade, and the direct interaction between an E3 ubiquitin ligase and the cognate substrate proteins is required for completing the last step of ubiquitination [54,55]. The observed interaction between HBV capsid and the ubiquitinated Alix indicated that an E3 ubiquitin ligase exists, which could promote not only Alix ubiquitination but also naked capsid secretion. Numerous studies have shown that NEDD4 family E3 ubiquitin ligases play critical roles in the ESCRT/MVB sorting pathway and enveloped virus secretion [13,56], including a previous report that Nedd4 regulated HBV virion secretion through interacting with a late (L)-domain like PPAY motif of HBc protein [37]. However, we found that ectopic expression of Nedd4 did not affect HBV naked capsid secretion in cells co-transfected with HBc (S10 Fig, lane 2 vs 1). Thus, we also included other NEDD4 family members in the test, including Nedd4L, WWP1, WWP2, ITCH/AIP4, Smurf1, Smurf2 and BUL1 [57], and the screening scored AIP4 as a facilitator of empty naked capsid secretion (S10 Fig, lane 9 vs 6). Further validation assays showed that the ectopically expressed AIP4 promoted naked capsid secretion without affecting the levels of intracellular HBc and capsid (Fig 6A), and knockdown of endogenous AIP4 by siRNA significantly reduced naked capsid secretion without downregulating intracellular HBc/capsid (Fig 6B).

Next, we examined the effect of AIP4 on the egress of naked capsid with different viral genome contents. Firstly, we analyzed HBV replication and secretion in HepG2 cells co-transfected with the envelope-null HBV plasmid duet pTREHBVDES/pTet-off with and without AIP4. The results showed that AIP4 inhibited intracellular HBV expression and replication due to a primary reduction of HBV RNA (Fig 6C, top panels), indicating that AIP4 may suppress HBV transcription or RNA stability. However, the levels of extracellular total and DNA-containing naked capsids were comparable to the control (Fig 6C, bottom panels). Such observation was more striking in 293T cells where AIP4 overexpression dramatically increased total and DNA-containing naked capsid egress despite of the reduction of intracellular viral capsid production (S11A Fig). These results inferred that AIP4 also promotes the secretion of HBV DNA-containing naked capsid, while the mechanism underlying the inhibitory effect of AIP4 on HBV transcription awaits further investigation. Furthermore, we assessed the effect of AIP4 on HBV RNA-containing naked capsid secretion. The envelope-null and priming-defective HBV mutant plasmid pCMVHBV-Y63DΔS was employed for arresting viral replication at pgRNA-containing capsid step. As shown in Figs 6D and S11B, AIP4 overexpression increased the levels of HBV total and pgRNA-containing naked capsids in the supernatant of HepG2 and 293T cells, although intracellular HBV nucleocapsid production was reduced primarily due to pgRNA reduction. Collectively, the above results demonstrate that AIP4 promotes naked capsid egress irrespective of their HBV genome contents.

### Mapping the functional domain of AIP4 responsible for promoting naked capsid egress

**Fig 7:**
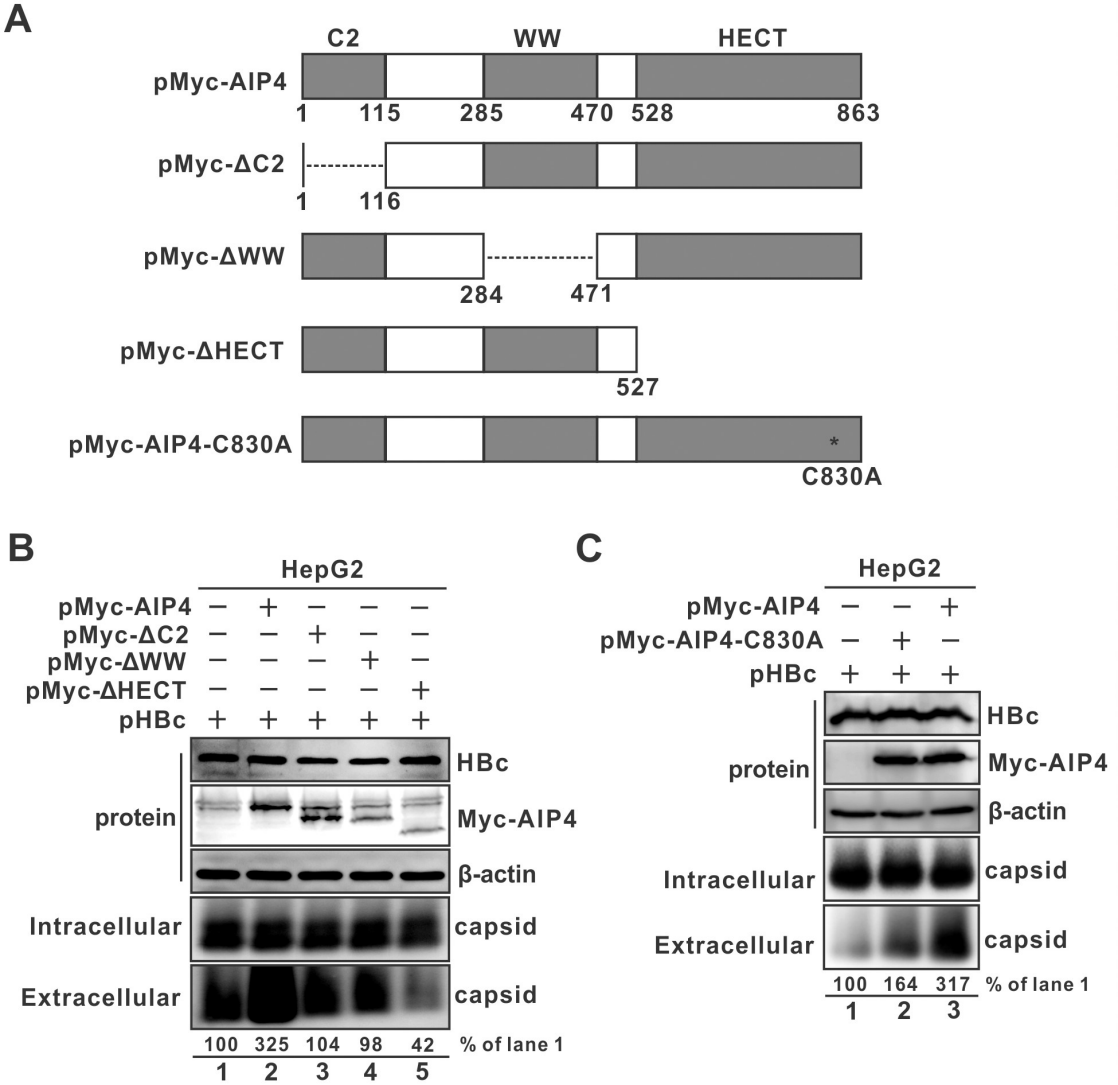
Mapping the domains of AIP4 responsible for promoting naked capsid secretion. (A) Schematic representation of wt AIP4 and mutants. The domain architecture of AIP4 with the C2, WW and HECT domains is depicted. The amino acid (aa) positions are labeled with numbers. The enzymatically inactive mutant (C830A) is marked with an asterisk. (B) The protein integrity is crucial for AIP4 to promote naked capsid secretion. HepG2 cells were co-transfected with pHBc and the control vector or the plasmid expressing full length AIP4, ΔC2, ΔWW, or ΔHECT for 3 days, the expression of transfected AIP4 and HBc was detected by Western blot, β-actin served as a loading control, intracellular and extracellular capsids were revealed by particle gel immunoblot. (C) The E3 ubiquitin ligase activity of AIP4 is partially responsible for AIP4-mediated enhancement of naked capsid secretion. HepG2 cells were co-transfected with pHBc and the control plasmid or full-length AIP4, or C830A mutant, for 3 days, followed by Western blot analyses of intracellular AIP4 and HBc proteins and particle gel assay of intracellular and extracellular capsids.

AIP4 possesses an N-terminal C2 domain, four internal WW domains, and a C-terminal HECT (Homologous to E6AP C-terminus) catalytic domain which mediates ubiquitin transfer to substrates [58]. To identify the functional domain of AIP4 responsible for promoting naked capsid egress, AIP4 domain-deletion clones and HECT domain E3 ligase inactive mutant C830A were constructed as schematically illustrated in Fig 7A and tested for their effects on naked capsid egress upon co-transfection with HBc. Notably, removal of each domain of AIP4 abolished the stimulatory effect of AIP4 on naked capsid egress, and the HECT-domain deletion even inhibited naked capsid secretion compared to the control perhaps due to a dominant-negative effect (Fig 7B). Surprisingly, the enzymatically inactive C830A mutant only partially attenuated the AIP4-stimulated naked capsid egress (Fig 7C). These results indicate that the protein integrity is crucial for AIP4 to promote naked capsid egress, and in terms of the HECT domain, the domain itself is more important for AIP4-mediated enhancement of naked capsid egress than its E3 ligase activity.

### AIP4 interacts with Alix and ubiquitinates Alix

**Fig 8:**
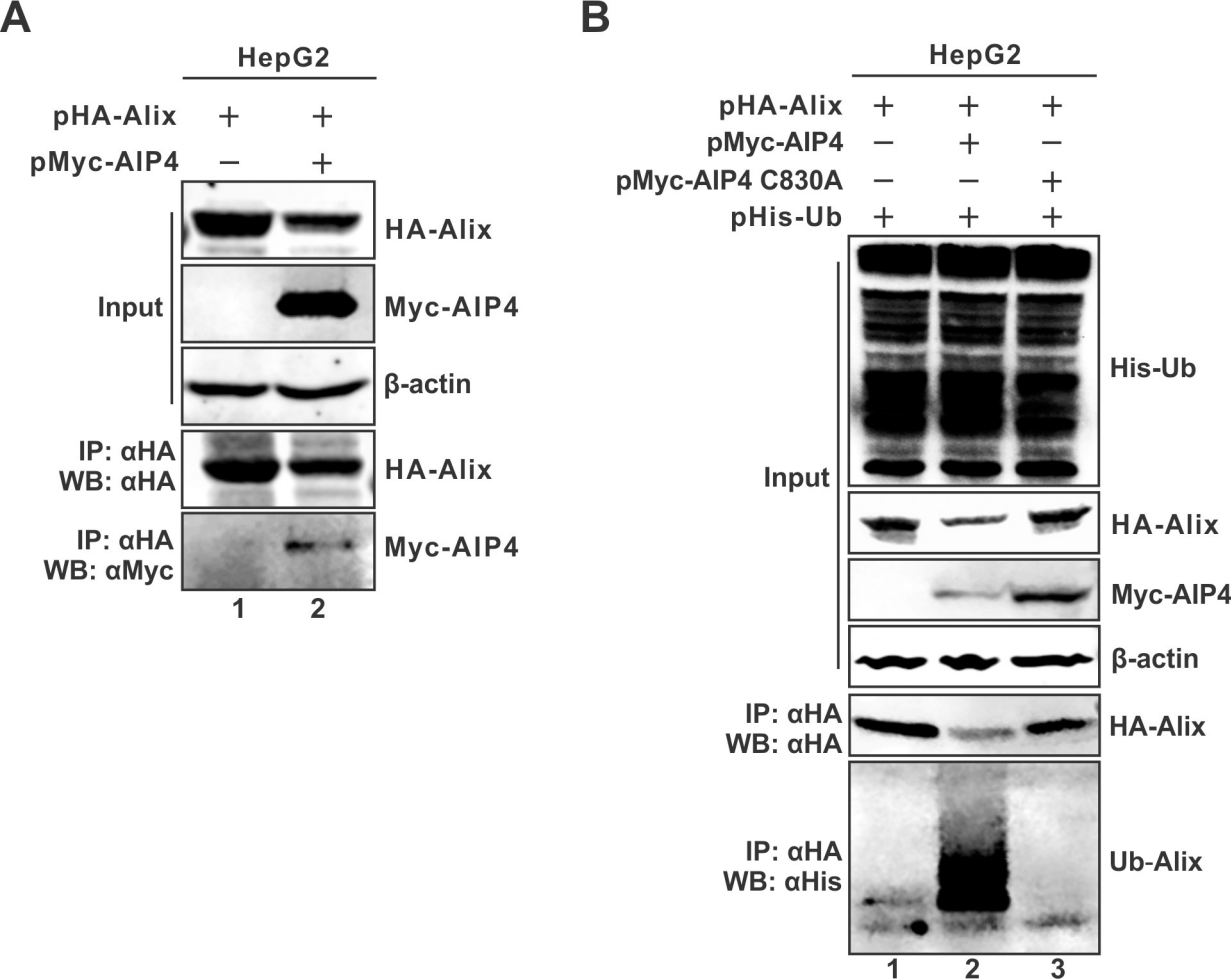
AIP4 interacts with Alix and promotes Alix ubiquitination. (A) AIP4 is co-immunoprecipitated with Alix. HepG2 cells were co-transfected with HA-Alix and Myc-AIP4 or control vector for 3 days. The expression of transfected HA-Alix and Myc-AIP4 was detected by Western blot. Co-immunoprecipitation of HA-Alix was performed, and the immunoprecipitated HA-Alix and Myc-AIP4 were detected by Western blot. (B) Alix is ubiquitinated by AIP4. HepG2 cells were co-transfected with HA-Alix and His-Ub plus control vector or Myc-AIP4 or Myc-AIP4 C830A for 3 days. The expression of transfected proteins was detected by Western blot. Co-immunoprecipitation of HA-Alix was performed, and the immunoprecipitated HA-Alix and His-Ub-ubiquitinated Alix (Ub-Alix) were detected by Western blot.

Given that both Alix and AIP4 promote naked capsid egress, it is of interest to assess a potential interaction between AIP4 and Alix by co-IP assay. The results showed that Myc-AIP4 co-immunoprecipitated with HA-Alix (Fig 8A). Moreover, confocal microscopy analysis revealed a co-localization of the above two proteins in the cytoplasm (S12 Fig), further demonstrating that AIP4 interacts with Alix.

Next, we asked whether AIP4 could ubiquitinate Alix. To address this question, Alix was co-expressed with wt AIP4 or the catalytically inactive C830A mutant in the presence of His-Ub, followed by co-IP assay. The results showed that the ubiquitination level of Alix was significantly upregulated by wt AIP4 but not the C830A mutant (Fig 8B). Hence, the above results demonstrate that AIP4 is an E3 ubiquitin ligase for Alix.

It is worth noting that the overexpression of wt Myc-AIP4 but not the C830A mutant led to a notable reduction of co-expressed HA-Alix (Fig 8), which might be due to the Myc-AIP4-mediated HA-Alix ubiquitination and subsequent proteasomal degradation, and/or the similar mechanism for Myc-AIP4-mediated inhibition of plasmid-based HBV transcription (S11 Fig). The underlying mechanism needs to be explored in future studies.

### AIP4 and Alix interdependently promote naked capsid egress

**Fig 9:**
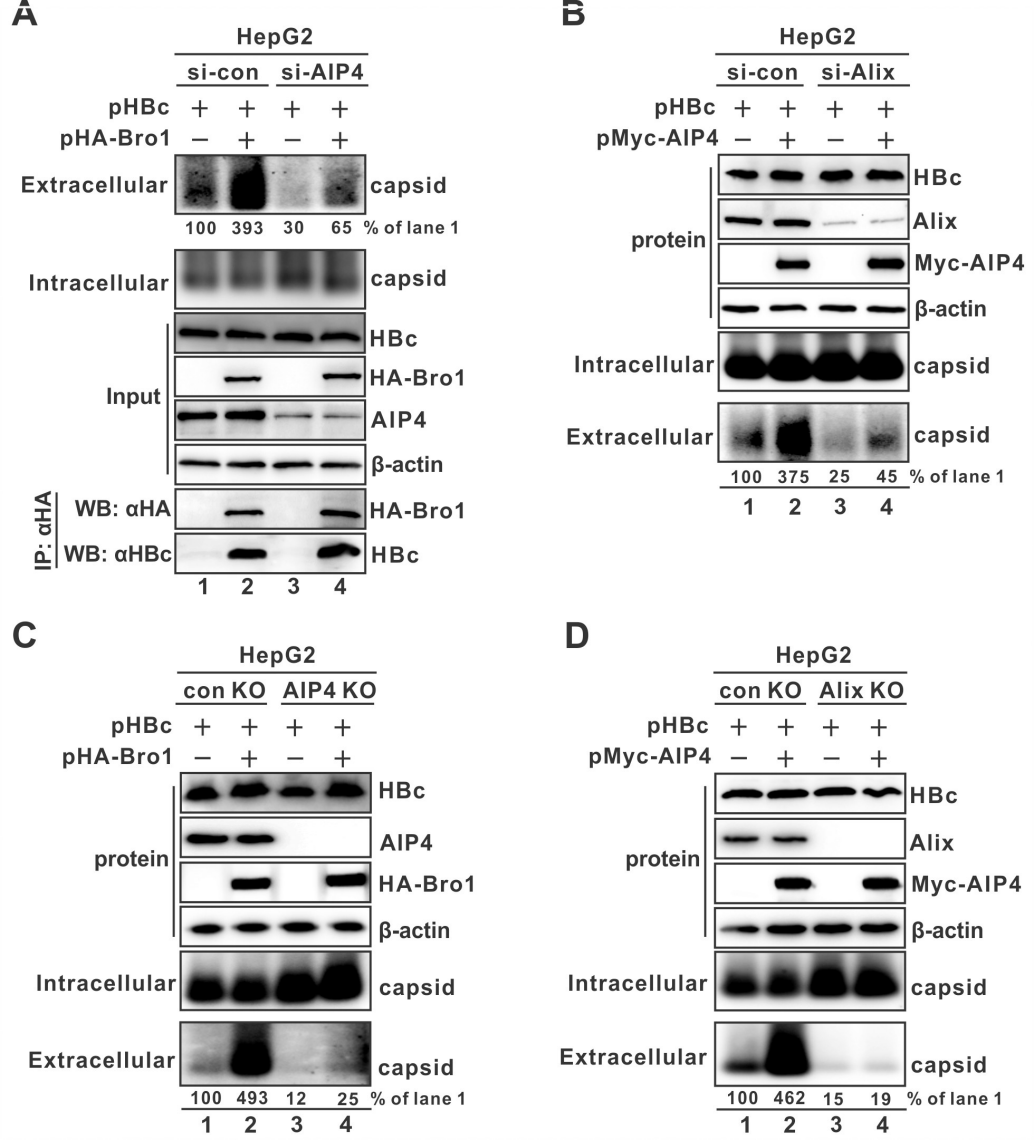
AIP4 and Alix interdependently promote naked capsid secretion. (A) Knockdown of AIP4 inhibited Alix Bro1-mediated naked capsid secretion without affecting the intracellular Bro1-HBc interaction. HepG2 cells were transfected with si-con or si-AIP4 for 24 h, followed by co-transfection with pHBc and control vector or HA-Bro1. Intracellular and extracellular capsids were detected by particle gel immunoblot (upper panels). The expression of transfected HBc, HA-Bro1, and endogenous AIP4 was detected by Western blot. Co-immunoprecipitation of HA-Bro1 was performed, and the immunoprecipitated HA-Bro1 and HBc were detected by Western blot. (B) Knockdown of Alix blocked AIP4-mediated enhancement of naked capsid secretion. HepG2 cells were transfected with si-con or si-Alix for 24 h and then transfected with pHBc plus control vector or Myc-AIP4 for 3 additional days. The expression of transfected HBc, Myc-AIP4, and endogenous Alix was analyzed by Western blot. Intracellular and extracellular capsids were detected by particle gel immunoblot. (C) Knockout of AIP4 attenuated Alix Bro1-mediated naked capsid secretion. HepG2 control knockout (con KO) and AIP4 knockout (AIP4 KO) cells were co-transfected with pHBc and control vector or pHA-Bro1 for 3 days. The expression of transfected HBc, HA-Bro1, and endogenous AIP4 was analyzed by Western blot. Intracellular and extracellular capsids were detected by particle gel immunoblot. (D) Knockout of Alix abolished AIP4-enhanced naked capsid secretion. HepG2 con KO and Alix knockout (Alix KO) cells were transfected with pHBc and control vector or pMyc-AIP4 for 3 days. The expression of transfected HBc, Myc-AIP4, and endogenous Alix were analyzed by Western blot, β-actin served as a loading control. Intracellular and extracellular capsids were detected by particle gel immunoblot.

Since both Alix and AIP4 promote naked capsid egress and AIP4 ubiquitinates Alix, it is interesting to investigate whether AIP4 is a cofactor to assist Alix in mediating naked capsid egress. We thus assessed the potential interactions among Alix Bro1 domain, HBc, and AIP4, as well as the level of Bro1-mediated naked capsid secretion by manipulating the expression level of AIP4. As shown in Fig 9A, knocking down AIP4 did not alter the interaction between Alix Bro1 and HBc (bottom panels, lane 4 vs 2), indicating that AIP4-mediated naked capsid secretion is not through targeting the interface between Alix and capsid. However, the knockdown of AIP4 significantly reduced the basal level of naked capsid secretion (top panel, lane 3 vs 1) and Bro1-stimulated naked capsid secretion (top panel, lane 4 vs 2). On the other hand, knocking down Alix dramatically diminished the stimulatory effect of AIP4 on naked capsid secretion (Fig 9B, lane 4 vs 2).

To further assess the above observed cooperative role of Alix and AIP4 in naked capsid secretion, the expression of Alix or AIP4 in HepG2 cells was knocked out by CRISPR/Cas9, and the knockout effect on AIP4- or Bro1-stimulated effect on naked capsid secretion was examined, respectively. As shown in Fig 9C and 9D, knockout of either AIP4 or Alix significantly reduced the basal level of supernatant naked capsid (lanes 3 vs 1) and resulted in an almost complete loss of the stimulatory effect of overexpressed Bro1 or AIP4 on naked capsid egress (lanes 4 vs 2), respectively. It is worth noting that there was a minimal residual level of supernatant naked capsid under the condition of AIP4 or Alix knockout (lanes 3–4), the reason is unknown but may be due to an AIP4/Alix-independent mechanism. Nonetheless, the above results clearly demonstrated that AIP4 and Alix interdependently promote naked capsid egress.

### AIP4-mediated ubiquitination of Alix is dispensable for naked capsid egress

**Fig 10:**
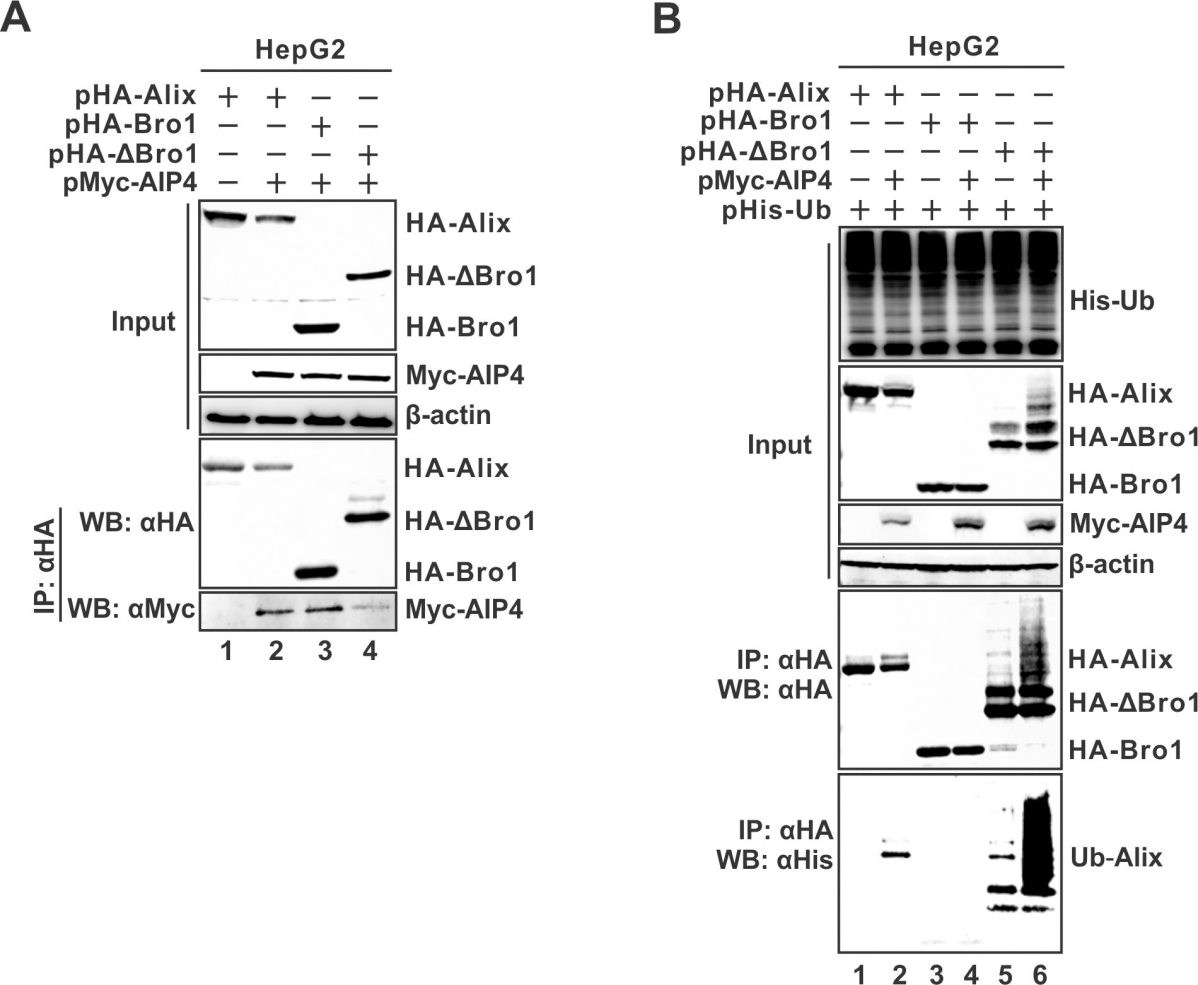
AIP4-mediated Alix ubiquitination is dispensable for naked capsid secretion. (A) AIP4 mainly binds to the Bro1 domain of Alix. HepG2 cells were co-transfected with Myc-Aip4 and full-length HA-Alix, or HA-Bro1, or HA-ΔBro1 for 3 days. The expression of transfected Myc-AIP4 and full-length and truncated HA-Alix was detected by Western blot. Co-immunoprecipitation of HA-Alix was performed, and the immunoprecipitated full-length and truncated HA-Alix and Myc-AIP4 were detected by Western blot. (B) AIP4-mediated ubiquitination of Alix is independent of the Bro1 domain. HepG2 cells were co-transfected with His-Ub and HA-Alix, or HA-Bro1, or HA-ΔBro1 together with the control vector or Myc-AIP4 as indicated for 3 days. The His-Ub-ubiquitinated proteins, full-length and truncated HA-Alix, and Myc-AIP4 were detected by Western blot, β-actin served as loading control. Co-immunoprecipitation of HA-Alix was performed, and the immunoprecipitated full length and truncated HA-Alix and His-Ub-ubiquitinated Alix (Ub-Alix) were detected by Western blot.

Finally, we assessed whether the AIP4-catalyzed Alix ubiquitination is functionally required for naked capsid egress. Since the Bro1 domain is indispensable for Alix-mediated naked capsid egress, we thus examined whether AIP4 could interact with the Bro1 domain and promote its ubiquitination. The co-IP assay showed that AIP4 preferentially binds to the Bro1 domain rather than the rest part of Alix (Fig 10A, lane 4 vs 3). However, the in-cell ubiquitination assay demonstrated that Bro1 domain ubiquitination was undetectable at basal level or under AIP4 overexpression (Fig 10B, lanes 3–4); in marked contrast, ubiquitination of the AlixΔBro1 moiety was detected and it was further enhanced by AIP4 (Fig 10B, lanes 5–6). These results suggest that the interaction of AIP4 with Alix, rather than AIP4-catalyzed Alix ubiquitination, enables HBV naked capsid egress.

### Discussion

Hepadnaviral double-stranded DNA-containing, pgRNA-containing, or even empty capsids are capable of being enveloped and secreted as extracellular virions, and the viral envelope proteins can be secreted in surplus as subviral particles devoid of exterior capsid [7,8]. In addition to the enveloped viral particles, the supernatant of HBV-producing hepatoma cell lines contains a considerable amount of non-enveloped naked capsids as evidenced by electron microscopy, density gradient fractionation, particle gel assay, or Western blot [22–24,40–47]. However, this obvious phenomenon has been overlooked for long as the naked capsid had been thought to be a by-product of HBV replication or an artifact in cell cultures. Thus far, there are only several reported studies on the regulation of naked capsid secretion *in vitro* [22–24,59].

**Fig 11:**
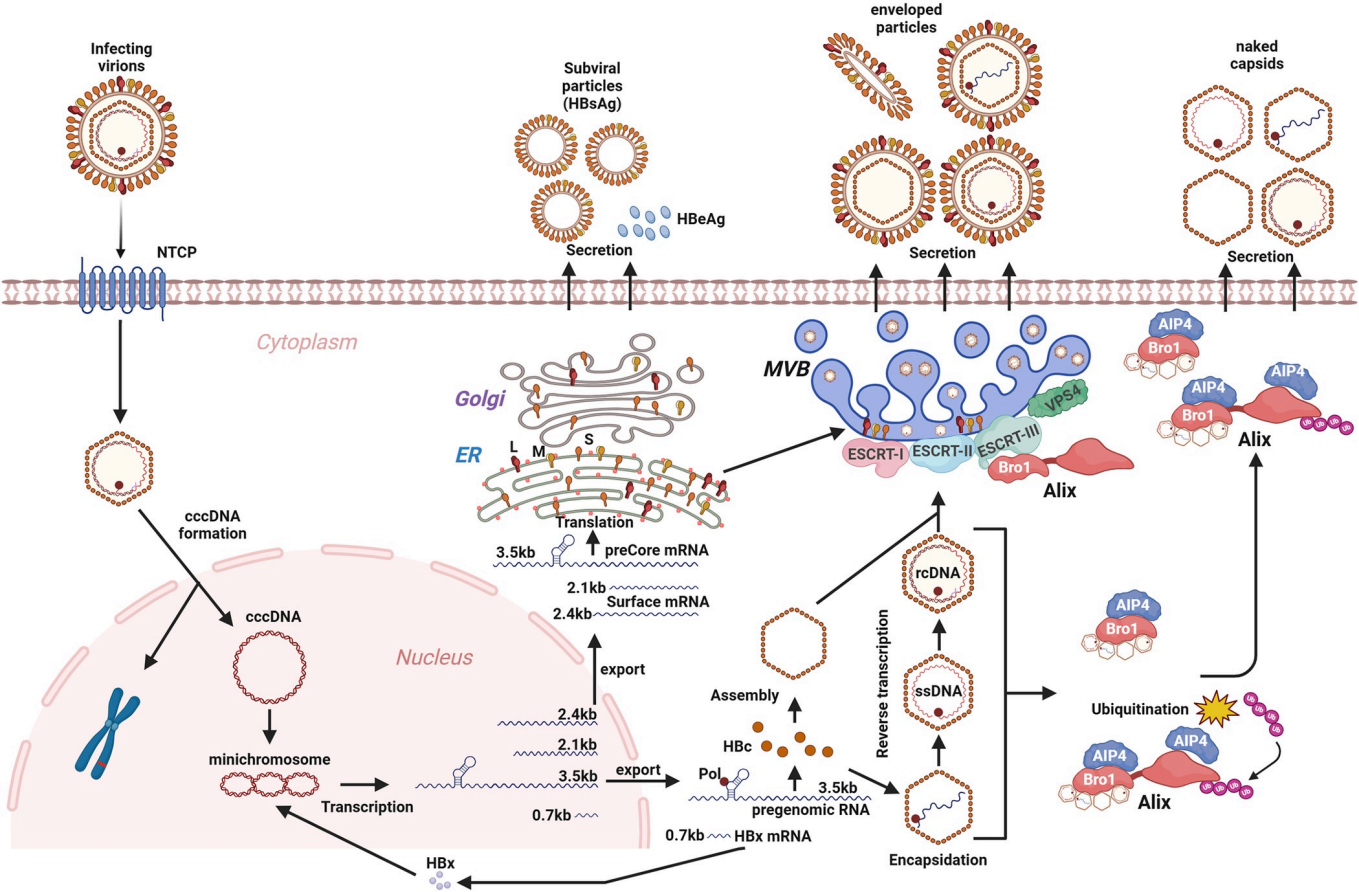
The proposed model for naked capsid secretion. See Introduction for a brief description of the canonical HBV replication cycle. The newly synthesized HBeAg and spherical HBsAg are secreted through the ER-Golgi constitutive secretory pathway. The filamentous HBsAg, empty capsid, pgRNA-containing capsid, and rcDNA-containing capsid can be enveloped and secreted via cellular ESCRT/MVB pathway. The ubiquitinated ESCRT accessory protein Alix recognizes intracellular HBV capsid through its Bro1 domain and promotes naked capsid egress irrespective of their HBV genome contents. The ubiquitination of Alix is mediated by the NEDD4 family E3 ubiquitin ligase AIP4. Mechanistically, AIP4 and Alix interdependently promote naked capsid egress, whereas AIP4 preferentially binds to the N-terminal Bro1 domain of Alix. However, the Bro1 domain of Alix is non-ubiquitinated, but the rest part of Alix can be ubiquitinated by AIP4, suggesting that the interaction of AIP4 with Alix, rather than AIP4-catalyzed Alix ubiquitination, drives HBV naked capsid egress. Created with BioRender.com.

In this study, we have comprehensively characterized naked capsid and provided multiple, complementary lines of evidence to demonstrate that the secretion of naked capsid is the result of a series of finely tuned interactions between the virus and host. First, through analyzing multiple HBV- and DHBV-producing cell systems, naked capsids were ubiquitously detected in the culture fluids regardless of the type of viral nucleic acid content or cell background (Figs 1 and S1), suggesting that the release of naked capsid into supernatant is a common event of *in vitro* hepadnaviral replication cycle. Second, naked capsid was detected in the sera of HBV-infected humanized mice but not in the sera of HBV transgenic mice or DHBV-infected ducklings (S2A and S3 Figs), indicating that the *in vivo* secreted naked capsid can be rapidly eliminated by the host immune system, likely the HBcAb-mediated phagocytosis (S2B Fig). It is worth noting that an early study could not detect free HBcAg in HBcAb-negative HBV carriers [60], the possible explanations include, but not limited to, 1) a low level of circulating naked capsid; 2) naked capsids and HBcAb form immunocomplexes and become undetectable by enzyme immune assays [61,62]. Third, by analyzing the Lysine residues on HBc protein, we found that HBc K96 is important for naked capsid secretion as K96R mutant cannot be released as naked capsid (Figs 2, S4 and S5), a phenotype without alteration of cell membrane integrity or cell viability but accompanied with the loss of ubiquitin on intracellular capsid (Figs 3 and S6). Further mutagenesis of K96 demonstrated that the interaction between ubiquitin and K96 is bridged through a ubiquitinated host protein(s) rather than the ubiquitination of K96 (Fig 4). Fourth, the host ESCRT-III accessory factor Alix, which has been shown to direct the egress of naked capsid in cell cultures, is associated with K96 in a ubiquitinated form (Figs 5, S8 and S9). Lastly, NEDD4 E3 ubiquitin ligase AIP4 facilitates naked capsid egress through binding to Alix but does not require AIP4-catalyzed Alix ubiquitination (Figs 6–10, and S10–S12). In alignment with prior research, our study provides further evidence suggesting that HBV naked capsids are nonlytically released from cells via a yet inadequately defined secretory mechanism. A model of naked capsid secretion is proposed for the following discussion (Fig 11).

HBc is a 21-KDa protein comprising an N-terminal domain (NTD) and an Arginine-rich C-terminal domain (CTD) linked by a short linker peptide. The CTD contains an array of major and minor phosphorylation sites, which undergo dynamic phosphorylation and dephosphorylation process to regulate capsid assembly, pgRNA encapsidation, reverse transcription, envelopment, and rcDNA genome uncoating [26–28,63–65]. Besides the extensively studied HBc phosphorylation, other PTMs of HBc and their roles in regulating HBV replication need to be further characterized [66]. HBc possesses two conserved Lysine residues, K7 and K96, and a recent mass spectrometry analysis has identified ubiquitinated K7 but not K96 [34]. However, a recent mutagenesis study and our results presented here clearly infer that the ubiquitination of K7 and its other potential PTMs, if any, are not essential for HBV to complete replication and secretion (Figs 2 and S4). In terms of K96, the K96R mutant did not affect intracellular HBV DNA replication in our study as previously reported [16,36], and it did not exhibit phenotype of HBV virion secretion on particle gel either (Fig 2). The lack of effect of K96R on HBV virion secretion has also been observed in one previous study [36], however, three other studies reported that K96R mutant blocked virion secretion [16,39,53]. The exact reason causing such discrepancy is unknown but may be due to differences among different HBV virion assays (particle gel, anti-HBs-IP and endogenous polymerase reaction, qPCR) and potentially different genetic backgrounds of cell lines and/or HBV isolates/variants. Moreover, the K96R mutant supported lower levels of transfection-derived HBV DNA replication in two of the three aforementioned studies [39,53], and the reduction of extracellular HBV DNA in the context of K96R mutation reported by another study was likely due to the loss of naked capsid particles, which contain the major portion of supernatant HBV DNA [16]. It is also worth noting that naturally occurring K96R mutant has been found in serum HBV DNA of chronic hepatitis B patients [39,67–70], which indicates that K96R may not be a lethal mutant for HBV virion secretion, at least in the probable chimeric capsid with wt HBc.

Since K96 is a potential ubiquitination site and it has been reported to interact with an endosomal E3 ubiquitin ligase Nedd4 to direct HBV virion formation and secretion, we thus reasoned that the K96R-associated loss of naked capsid egress might be a consequence of alteration of Nedd4-mediated HBc ubiquitination. Indeed, the co-IP assay demonstrated that wt HBc/capsid but not K96R was associated with ubiquitin, however, it was apparently not a direct ubiquitination of HBc, at least not polyubiquitination (Fig 3). Furthermore, other K96 substitutions (E, H, M, P and Q) showed a nice correlation of naked capsid egress-competent capsids with ubiquitin binding (Fig 4A and 4B). To our surprise, only the conservative mutations K96R and K96H lost ubiquitin binding and naked capsid secretion, the underlying reason is currently not known. Nonetheless, we could not rule out the possibility that a trace amount of HBc/capsid ubiquitination was below the detection limit of our assay. By utilizing the conjugation-deficient ubiquitin mutant G75/76A, we further confirmed that detected ubiquitin associated with wt HBc/capsid is carried by a ubiquitinated cellular protein(s). Thus, identifying such protein(s) would aid the mechanistic study of naked capsid secretion.

The heterogeneous viral nucleic acid content of naked capsid indicates a less strict intracellular sorting mechanism for naked capsid egress (Fig 1), but the exact ratio of different naked capsid particles needs to be determined. Interestingly, the ESCRT/MVB-mediated egress of HBV virion also exhibits less selectivity of intracellular capsid for envelopment except that the single stranded DNA-containing capsid is excluded from virion formation (Fig 11) [8,25,71]. Therefore, it is possible that the mechanism of naked capsid egress may partially overlap with virion’s (Fig 11). Previous studies have suggested that naked capsid egress is independent of the integral ESCRT/MVB secretory pathway, but an ESCRT-0 component HGS and an ESCRT-III-binding protein Alix could facilitate naked capsid egress in cell cultures [22–25]. Since HGS has been shown to promote naked capsid release in a ubiquitin-independent manner [24], Alix was thus prioritized for assessment of potential involvement in ubiquitin-associated naked capsid egress. Consistent with the previous study, the full-length Alix and its Bro1 domain alone promoted naked capsid egress, and the Bro1 domain was required for interacting with HBc (Figs 5A–5C and S8) [23]. Additionally, we found that K96 is responsible for the binding of HBc with Alix, as Alix cannot bind to the secretion-incompetent HBc mutants K96R and K96H (Fig 5C). The interaction between Alix and HBc likely occurs on the capsid surface, and in line with this, the structure modeling of HBV capsid revealed that K96 is exposed on the outer surface of HBc dimer and capsid [21,30,31]. However, further structural investigation is needed to determine the stoichiometry of Alix-capsid binding and the mechanism underlying how a K96R/H single mutant disrupts the association of Alix to capsid. Moreover, the potential effect from the neighboring residues of K96 may also be taken into consideration in the context of K96R/H mutation. Previous studies have shown that mutations of L95 or I97 of HBc could affect nucleocapsid formation, envelopment, and secretion [31,72–75]. Further studies are needed to investigate whether these sites contribute to the phenotypes of K96 mutagenesis and the ability of Alix to bind to capsid. Furthermore, the mechanism of Alix-mediated naked capsid secretion remains poorly understood. It has been speculated that the boomerang-shaped Bro1 domain of Alix directly acts on the naked capsid budding site on the plasma membrane by utilizing an inherent membrane-deforming ability of Bro1 to induce membrane rupture for naked capsid egress [23]. If this is the case, the Alix-induced membrane rupture should be mild and rapidly healed, as the membrane integrity assay did not show notable difference between cells expressing wt HBc and K96R mutant (S6 Fig). Interestingly, we found that Alix can be ubiquitinated, which may explain the HBc-associated ubiquitin as knockdown of endogenous Alix impaired the interaction between HBc and ubiquitin (Figs 5D–5E and S9). It has been reported that human Alix and its yeast homologue function as a highly conserved ubiquitin receptor for cargo sorting in ESCRT/MVB secretory pathway (Fig 11), and the Alix ubiquitin-binding or ubiquitination may regulate self-dimerization, which is responsible for interaction with ESCRT-III filaments on plasma membranes during HIV budding [76–79]. Therefore, it was of further interest to identify the E3 ubiquitin ligase responsible for Alix ubiquitination in the context of Alix-mediated naked capsid secretion.

NEDD4 E3 ubiquitin ligase family members usually possess one N-terminal C2 domain responsible for protein’s intracellular localization through interacting with phospholipids, three or four internal WW domains responsible for substrate recognition by binding to a PPxY motif, and one C-terminal HECT domain encoding the E3 catalytic activity, thus, NEDD4 family also belongs to the HECT class of E3 ligases [58]. The cellular MVB secretory pathway requires NEDD4 family members act as functional links between ESCRT and the cargo proteins containing the PPxY late (L) domain motif [58]. Numerous studies have demonstrated that NEDD4 proteins facilitate the budding of various enveloped viruses through ubiquitinating viral matrix proteins or host adaptor proteins in a PPxY motif-dependent fashion, including HIV, Ebola virus, vesicular stomatitis virus, Marburg virus, Rous sarcoma virus, etc. [56,80]. HBc encodes a L domain-like motif (129-PPAYRPPNAPI-139) that has been reported to recruit NEDD4 family member Nedd4, ubiquitin-binding adaptor γ2-adaptin, and ESCRT-I component TSG101 for virion maturation and egress through ESCRT/MVB [16,37,81]. In addition, the HBc L domain-like motif also contains a putative Alix-interacting YPXL motif (131-AYRPPNAPI-139), which matches the reported consensus sequence ΦYX0/2(P/Φ)X0/3(L/I) (where Φ denotes a hydrophobic residue and X denotes amino acid spacers of different lengths) [82,83]. It has been reported that Alix binds to the YPXL motif of HIV-1 Gag and recruits Nedd4 to facilitate virus release, a mechanism that involves Alix ubiquitination [84]. Considering that HBV releases naked capsid via an HBc ubiquitination-independent but Alix-dependent way as described above, we thus speculated that a NEDD4 ligase(s) may facilitate naked capsid egress via ubiquitinating Alix. By screening all the known NEDD4 family members for their effect on naked capsid egress, AIP4 (aka ITCH) was scored as a hit that promotes naked capsid egress upon ectopic expression, and it has been validated through further assessing the dose-dependent effect and knockdown effect (Figs 6A, 6B and S10). Moreover, the AIP4-mediated enhancement of naked capsid secretion was irrespective of viral DNA, RNA, or empty genome content (Fig 11), but AIP4 overexpression significantly inhibited HBV replication primarily through reducing the level of viral pgRNA transcripts (Figs 6C, 6D and S11). A similar dual effect has also been observed in a previous study on HGS [24]. Considering that the pgRNA transcription is governed by a CMV-IE promoter (wt or tet-inducible) in the HBV constructs used in our experiments (Fig 6C and 6D), it is possible that AIP4 may target a host protein(s) maintaining CMV-IE promoter activity or HBV pgRNA stability for ubiquitination and proteosome degradation. More importantly, we found that the AIP4-mediated enhancement of naked capsid egress relies on its E3 ligase activity encoded by the HECT domain. While the enzymatically inactive mutation (C830A) within the HECT domain significantly impaired the AIP4-mediated enhancement of naked capsid egress, the HECT domain deletion mutant even exhibited a dominant-negative effect on naked capsid egress (Fig 7), indicating a pivotal role of both the activity and integrity of HECT domain.

We thus hypothesized that Alix could be the substrate of AIP4 in promoting naked capsid egress in light of the role of Alix. Indeed, Alix was found to interact with AIP4 by both the co-immunoprecipitation assay and fluorescence colocalization microscopy analysis (Figs 8A and S12); and AIP4 could significantly up-regulate the ubiquitination level of Alix (Fig 11), whereas the enzymatically inactive C830A mutant failed to do that and even exhibited a dominant negative effect (Fig 8B). While the AIP4-Alix binding has been reported previously [85], to the best of our knowledge, our study provides the first evidence on AIP4-mediate Alix ubiquitination. Since the Bro1 domain of Alix is sufficient for naked capsid egress, we then examined whether the Bro1 domain can be ubiquitinated by AIP4. We found that AIP4 interacts with Alix at both the Bro1 domain and beyond (Figs 10A and 11). Interestingly, AIP4 overexpression upregulated the ubiquitination of wt Alix and ΔBro1 mutant, but not the Bro1 domain, and no ubiquitination was detected on Bro1 domain only (Figs 10B and 11), indicating that Alix ubiquitination is not absolutely required for Alix-mediated naked capsid egress and its enhancement by AIP4 (Fig 11). However, considering that the catalytic activity of AIP4 is needed for the enhancement of naked capsid egress, other co-factor(s) involved in naked capsid egress that can be ubiquitinated by AIP4 may exist and contribute to the above observed capsid-associated ubiquitin signal. Another possibility is that the enzymatically active site C830 of AIP4 is also responsible for AIP4-Alix interaction. In addition, we demonstrated that AIP4 and Alix interdependently direct the egress of naked capsid (Fig 9). Collectively, the above results suggest that AIP4 interacts with Alix to enable HBV naked capsid egress in an Alix ubiquitination-independent manner.

Even with the additional information derived from this study, the mechanism of HBV naked capsid secretion remains largely elusive, particularly the lack of a coherent pathway upstream and downstream of the AIP4/Alix duet. The potential interplay between Alix and HGS in naked capsid egress awaits further investigation [24], and the AIP4 and Alix knockout data indicates that there may be a minor naked capsid egress mechanism independent of AIP4 and Alix (Fig 9). Moreover, Alix is also known to be important for the release of exosomes and it serves as an exosome marker [86], it remains to be tested whether manipulating exosome biogenesis could affect the Alix-mediated naked capsid release. Exosomes are derived from the intracellular multivesicular late endosomes and lysosomes [87]. It has been reported that the early endosome formation or a complete and functional autophagy pathway is not required for naked capsid egress, but the naked capsid egress-promoting Alix and Bro1 domain appear to coincidently induce the upregulation of autophagosome marker LC3-II, and the autophagosome formation factors complex Rab33B-Atg5/12/16L1 favors capsid assembly [59]. The probable involvement of autophagic vesicles in the nonlytic release of naked viruses including poliovirus and astrovirus has been indicated in previous studies [88–90]. Alternatively, a previous study on HGS-mediated naked capsid egress speculated that the ectosomes or microvesicles, another class of extracellular vesicles (EVs) directly assembled and released from the plasma membrane, may be involved in naked capsid egress based on a C. elegans study showing the requirement of HGS for efficient ectosome formation [24,91]. In line with this, AIP4 has also been reported to play an important role in facilitating the incorporation of host cargo proteins into ectosomes [92]. Therefore, it remains to be determined whether these Alix- and/or AIP4-related intracellular and extracellular vesicles play any role in HBV naked capsid secretion. Nonetheless, it should be noted that the extracellular HBV naked capsids are seemingly envelope-free. If naked capsid is ejected into the extracellular milieu via EVs, then an immediate de-envelopment of EVs is required to efficiently release the naked capsid. In addition, it is also of interest to know why HBV capsid cannot be secreted in an exosome-like, quasi-enveloped particle like hepatitis A and E viruses do [93,94].

Another lingering question is whether the release of HBV naked capsid has any biological effect(s) on the infected host and/or the virus. From the host’s perspective, expelling the non-infectious naked capsids from infected cells may represent a host antiviral mechanism through prematurely stopping HBV virion morphogenesis, and the expelled naked capsids can be captured by the professional antigen presenting cells, including B cells, macrophages, and dendritic cells, to induce anti-HBc immunities. This notion is consistent with the rapid development and stable maintenance of high HBcAb titer during the natural history of HBV infection [48]. On the other hand, HBV or hepadnaviruses may use the circulating naked capsids as a decoy to help the virions to escape from host immunological control as the surface antigen surplus does. In line with this, a recent study observed that serum naked capsids exist in the form of capsid-antibody complexes in chronic hepatitis B patients [62]. Interestingly, the recently discovered nackednaviruses, a group of non-enveloped hepadnavirus-like fish DNA viruses, have been calculated to share a common ancestor with hepadnaviruses >400 million years ago [95]. These nackednaviruses possess structural conservation with HBV-like capsid proteins as well as a nonlytic yet unclear mechanism of capsid secretion [95,96]. In these regards, HBV and other hepadnaviruses might have inherited the ability of being secreted as naked capsid from their non-enveloped ancestor despite that they had evolved to have an enveloped life cycle. Hence, it would be interesting to employ nackednaviruses as model viruses to study this ancient mechanism of naked capsid egress.

Taken together, our study sheds new light on the molecular characteristics of extracellular hepadnaviral naked capsids and their secretory mechanism. The fine mechanisms by which Alix and AIP4 cooperate to promote HBV naked capsid egress are awaiting further investigation. Studying the noncanonical HBV subviral particles in the extracellular milieu will aid our understanding of the complexed viral life cycle, virus-host interaction, and the potential development of new antiviral strategies.

## Materials and methods

### Ethics statements

DHBV-positive duck serum was collected from a previous study [97]. Serum from HBV transgenic mice was leftover sample from previous study and provided as a gift by Dr. Michael Robek [98,99]. The HBV transgenic mice were housed in the Animal Resource Facility at Albany Medical College (AMC), and all experiments were done following protocols approved by the AMC Institutional Animal Care and Use Committee. Previously collected HBV-positive serum from virally infected uPA/SCID mice reconstituted with humanized liver was as a gift from Dr. Maura Dandri [100]. The animal experiments in Dr. Dandri’s laboratory were performed in accordance with the European Union directive 2010/63/EU and approved by the ethical committee of the city and state of Hamburg in accordance with the ARRIVE guidelines.

### Cell lines

HepG2, Huh7, 293T, and LMH cells were cultured in DMEM/F12 medium (Corning) supplemented with 10% fetal bovine serum, 100 U/ml penicillin and 100 μg/ml streptomycin. The HepG2-derived tetracycline (tet)-inducible HBV stable cell lines HepDE19 and HepDES19 were established previously, and maintained in the same way as HepG2, but with the addition of 1 μg/ml tet [40,101]. To induce HBV replication and virus particle secretion in HepDE19 or HepDES19 cells, tet was withdrawn from the culture medium, and the cells were cultured for the indicated time periods. The LMH-D2 cell line was derived LMH from chicken hepatoma cells by stable transfection of a pCMVDHBV plasmid constitutively transcribing the functional DHBV pgRNA and mRNAs for all the structural proteins, and it secrets DHBV virion into supernatant [102,103]. Dstet5 is an LMH cell-derived tet-inducible duck hepatitis B virus (DHBV) stable cell line supporting the replication of an envelope protein-deficient DHBV genome, and it was cultured as previously described [104].

AIP4 and Alix knockout (KO) cell lines were generated through CRISPR-mediated genome editing of the corresponding gene loci. The AIP4 and Alix Cas9/sgRNAs plasmids and the scramble control plasmid were purchased from Genepharma. These plasmids were transfected into HepG2 cells individually. Two days after transfection, puromycin antibiotic (4 μg/ml) was added to allow for positive selection of transfected cells. The cells were diluted and seeded onto 96-well plates for the growth of single cell-derived cell colonies. The AIP4 or Alix KO phenotype was confirmed by Western blot and Sanger sequencing.

### Plasmids, siRNA, and transfection

HBV replication-competent plasmid pCMVHBV (genotype D) supports transcription of pgRNA under CMV-IE promoter and transcription of subgenomic mRNAs under authentic HBV promoters [105]. Plasmid pCMVHBVΔC contains a start codon mutation (ATG to CTG) of the core ORF in pCMVHBV backbone to block the expression of core, which was introduced by using Q5 Site-Directed Mutagenesis Kit (NEB). Plasmid pCMVHBV-Y63DΔS supporting HBV pgRNA encapsidation but without reverse transcription and expression of surface proteins was described in a recent study [25]. Plasmids pTREHBVDES and pTet-off (Clontech) used to generate HepDES19 cell line were described previously [40]. Plasmids pHBc and pHBe expressing wt HBc and HBeAg, respectively, were constructed previously [106]. Plasmids pHBc-K7R, pHBc-K96R, pHBc-K7/96R, pHBc-K96E, pHBc-K96H, pHBc-K96M, pHBc-K96P, pHBc-K96Q, and pHBe-K96R expressing corresponding HBc and HBeAg K7 and/or K96 mutants were constructed by using the Q5 Site-Directed Mutagenesis Kit. The dimeric DHBV plasmid construct p2DHBV was described previously [103,107].

Plasmids expressing HA-tagged full-length Alix (pHA-Alix), Bro1 domain of Alix (pHA-Bro1), and Bro1 domain deletion mutant of Alix (pHA-AlixΔBro1) were kindly provided by Dr. Reinhild Prange [23]. Plasmids expressing the YFP-tagged HECT domain-containing human NEDD4 family ubiquitin ligases including Nedd4, Nedd4L, BUL1, WWP1, WWP2, ITCH/AIP4, Smurf1, and Smurf2 were gifts from Dr. Paul Bieniasz [57]. The vectors for expressing C-terminally Myc-tagged WT ITCH/AIP4 (c-myc–ITCH-WT) and the enzymatically inactive ITCH/AIP4-C830A mutant (ITCH-C830A) were kindly provided by Dr. Ronald Harty and were described previously [108,109]; these two plasmids were temporarily renamed to pMyc-AIP4 and pMyc-AIP4-C830A in this study for convenience. Plasmid pMyc-AIP4 was then used as the template to generate AIP4 domain deletion clones including pMyc-ΔC2, pMyc-ΔWW, and pMyc-ΔHECT by using the Q5 Site-Directed Mutagenesis Kit. Plasmid HA-Ub expressing the N-terminally HA-tagged ubiquitin from the pCMV-Script backbone was described previously [110]. The ubiquitination-defective double mutant G75/76A was introduced into HA-Ub by using Q5 Site-Directed Mutagenesis Kit and designated as HA-Ub^G75/76A^. Plasmid His-Ub was generated by replacing the HA-tag sequence on HA-Ub with the 6× histidine-tag sequence through mutagenesis.

AIP4 siRNA was purchased from OriGene; Alix siRNA was purchased from Genepharma.

Transfection of cells with plasmids and/or siRNA was performed by using Lipofectamine 2000 (Invitrogen) according to the manufacturer’s manual.

### Cell membrane integrity assay

The transfected cells were subjected to CytoTox-ONE Homogeneous Membrane Integrity Assay (Promega) by measuring the release of lactate dehydrogenase (LDH) from cells with a damaged membrane according to the manufacturer’s instructions.

### Hepadnaviral nucleic acids analyses

HBV total RNA, encapsidated pgRNA, and core DNA were extracted and subjected to Northern and Southern blot assays, respectively, as previously described [111–113]. Hybridization signals were recorded on a phosphorimager screen and scanned by the Typhoon FLA-7000 imager (GE Healthcare).

### Hepadnaviral particle gel assay

The intracellular HBV and DHBV capsids were analyzed by capsid gel assay as previously described [45]. The extracellular viral particles (virions, subviral particles, and naked capsids) and their DNA and RNA contents were analyzed by particle gel assay according to our previous publications [43,114].

### Sucrose density gradient fractionation

Cell sample in 10-cm-dish was lysed in 3 ml lysis buffer containing 10 mM Tris-HCl (pH 8.0), 10 mM EDTA, 1% NP-40, and 2% sucrose at 37°C for 10 min. Cell debris and nuclei were removed by benchtop centrifugation at 12,000 rpm for 15 min at 4°C. The HBV cell culture supernatant sample (~15ml) was clarified by ultracentrifugation in a Bechman SW28 rotor at 10,000 rpm for 1 h at 4°C. Sucrose density gradients were formed by overlaying 3 ml of each 5%, 10%, 15%, 20%, 25%, 30%, 35%, 40%, 45%, 50%, and 55% (wt/vol) sucrose solutions in TNE buffer (10 mM Tris-HCl, 100 mM NaCl, and 1mM EDTA) and placed at 4°C for 10 h to form continuous gradient. Then, the above prepared cell lysate or supernatant solution was overlaid on the gradients. Samples were ultracentrifuged in a Beckman SW28 rotor at 24,000 rpm for 16 h at 4°C. Eighteen fractions, each approximately 2 ml in volume, were meticulously collected from the bottom of the ultracentrifuge tube using a butterfly needle. The sucrose density (g/cm^3^) of each fraction was measured by the Abbe Refractometer. Each fraction was subjected to HBV particle/capsid gel assay.

### Transmission electron microscopy (TEM)

The clarified cell lysate or cell culture fluid prepared as aforementioned was loaded onto a 20% sucrose cushion in ultracentrifuge tube, followed by ultracentrifugation at 38,000 rpm for 5 h in a Beckman SW41 Ti swinging-bucket rotor at 4°C, the pellet was resuspended in 1 ml TNE buffer on a rotisserie for overnight at 4°C and the undissolved debris was removed by centrifugation at 12,000 rpm for 10 min at RT, and then the supernatant was subjected to another round of ultracentrifugation to spin down virus particles without the sucrose cushion. The pellet was resuspended in 200 μl TNE buffer. For each sample, an aliquot was mixed with the negative staining solution and pipetted onto a freshly glow-discharged carbon-coated TEM copper grid. The prepared samples were observed using a Tecnai BioTwin transmission electron microscope, and digital images were captured with an AMT CCD camera.

### Western blot assay

Whole cell lysate samples prepared by Laemmli buffer were resolved in a 12% SDS-PAGE and proteins were transferred onto Immobilon PVDF-FL membrane (Millipore). The membranes were blocked with Western Breeze blocking buffer (Life Technologies) and probed with antibodies against HBc [115], AIP4 (12117, Cell Signaling Technology (CST)), Alix (92880, CST), ubiquitin (sc-166553, Santa Cruz), HA-tag (3724, CST), His-tag (12698, CST), Flag-tag (F1804, Sigma), and β-actin (sc-58673, Santa Cruz). Bound antibodies were revealed by IRDye secondary antibodies. The immunoblot signals were visualized and quantified with the Li-COR Odyssey system.

### HBV HBeAg and HBcAb detection

The HBeAg and HBcAb in culture fluid and/or serum samples were detected by HBeAg ELISA kit (E0317, Autobio) and HBcAb ELISA kit (E0319, Autobio) following the manufacturer’s manual, respectively.

### Immunofluorescence and confocal microscopy

Cells were fixed with 4% paraformaldehyde for 20 min and permeabilized with 0.5% Triton X-100 in PBS for 60 min at room temperature. Fixed cells were blocked with 10% FBS and 2% bovine serum albumin (BSA) for 60 min at room temperature and then incubated with anti-Myc antibody (CST) and anti-HA antibody (CST) diluted in PBS containing 10% FBS and 2% BSA for 2 h at room temperature. After being washed with PBS, the cells were stained with the corresponding Alexa Fluor 405 and 594 dye-conjugated secondary antibodies (Abcam) or Alexa Fluor 488 dye-conjugated secondary antibody (Life Technologies) for 60 min at room temperature. Cell nuclei were stained with 4′,6-diamidino-2-phenylindole (DAPI). Finally, the cells were washed with PBS and then subjected to confocal microscopy analysis under an Olympus FV1000MPE microscope with a 60× objective. Images were processed by using FV10-ASW 3.0 Viewer software.

### Coimmunoprecipitation assay

HepG2 or 293T cells were cotransfected with indicated plasmids for 6 days. The harvested cells were lysed on ice with cell lysis buffer containing 1% NP-40, 10 mM Tris-HCl (pH 7.5), 1 mM EDTA, 50 mM NaCl, protease inhibitor cocktail, and Benzonase. After centrifugation to remove the cell debris, the clarified cell lysates were incubated with EZview red anti-HA affinity gel (Sigma-Aldrich) or EZview red anti-Flag affinity gel (Sigma-Aldrich) at 4°C overnight with gentle rotation. The beads were spun down the next day and resuspended gently with cold low-salt wash buffer (50 mM Tris-HCl [pH 7.4], 5 mM EDTA, 150 mM NaCl, and protease inhibitors) three times at 4°C, followed by one wash with high-salt buffer (50 mM Tris-HCl [pH 7.4], 5 mM EDTA, 500 mM NaCl, and protease inhibitors) and a desalting step with 20 mM Tris-HCl, pH 7.4. The washed beads were resuspended in Laemmli buffer without dithiothreitol (DTT) and spun down by centrifugation, the supernatant was collected, DTT was added to reach final concentration of 0.1 mM. The sample was boiled and subjected to Western blot assay.

### Statistical analysis

Data are provided as the mean ± standard deviation (SD). Statistical significance was considered with p value less than 0.05 (* p<0.05), and calculations and graphs were generated using GraphPad Prism 9.0.

## Supporting information

S1 Fig Sucrose density fractionation of HBV particles in culture fluid. The cytoplasmic lysate and supernatant of induced HepDE19 cells (tet-, day 8) were ultracentrifuged in the 5–55% sucrose gradient, followed by the collection of fractions from the bottom of the gradient. Each fraction was analyzed for sucrose density using a refractometer and subjected to particle gel immunoblot assay to detect cytoplasmic capsids, extracellular enveloped particles, and naked capsids. Note that the intracellular and extracellular capsids exhibit a similar density range.
(TIF)

S2 Fig Detection of HBV naked capsid in the sera of HBV transgenic mice and HBV-infected humanized mice. The induced HepDE19 cell culture fluid and indicated animal sera were subjected to (A) particle gel assay to detect HBV capsids and capsid-associated DNA by immunoblot and hybridization, respectively; and (B) HBcAb ELISA (mean ± SD, n = 3; ***p<0.001).
(TIF)

S3 Fig Detection of DHBV naked capsid in the serum of a virally infected duckling and supernatant of DHBV-replicating cells. DHBV-positive duck serum and supernatant of DHBV-transfected LMH cells and DHBV stable cell lines (dstet5 cells and LMH-D2 cells) were subjected to particle gel assay, virion, and naked capsid were revealed by hybridizing their viral DNA content with (-) strand-specific DHBV riboprobe.
(TIF)

S4 Fig HBc K96R mutation blocks “empty” naked capsid secretion. Plasmid expressing wt HBc or each mutant (K7R, K96R, K7/96R) was transfected into HepG2 cells for 3 days, followed by Western blot analysis of wt and mutant HBc proteins, and particle gel immunoblot assay of intracellular and extracellular capsids.
(TIF)

S5 Fig TEM analysis of HBV naked capsids. HepG2 cells were transfected with wt HBc or HBc-K96R mutant for 5 days. The intracellular (A, C) and extracellular (B, D) capsid particles were prepared as described in Materials and Methods and subjected to TEM visualization. Scale bar: 100 nm.
(TIF)

S6 Fig HBc transfection does not affect cell membrane integrity. HepG2 cells were transfected with the control vector or indicated wt or mutant HBc expression vector for 5 days, the release of LDH was measured by CytoTox-ONE Homogeneous Membrane Integrity Assay, and the relative cell viability values were calculated according to the manufacturer’s manual and plotted as the percentage of the value from control samples (mean ± SD, n = 3; ns: not significant).
(TIF)

S7 Fig The precore (pC) K96R mutation does not affect pC protein expression or HBeAg secretion. (A) Schematic representation of HBV C (HBc), pC, and HBeAg proteins. The aa positions are labeled with numbers, the first aa of C is set as position 1 and the position of K96 in each protein is marked with an asterisk. The pC protein possesses the same aa sequence with HBc plus the N-terminal 29 aa extension including a 19 aa signal peptide. The signal peptide sequence of pC is co-translationally cleaved, giving rise to a p22 intermediate, which is further processed by Furin peptidase in Golgi to remove the C-terminal domain and secreted as HBeAg. (B) HepG2 cells were transfected with plasmid expressing wt C, or wt pC, or pC-K96R mutant for 3 days. The expression of C, pC (p22), and pC-K96R was analyzed by Western blot, β-actin served as a loading control, intracellular capsids were revealed by capsid gel immunoblot. (C) HBeAg in the supernatant of above transfected cells was detected by ELISA and plotted as percentage of the pC-transfection positive control (mean ± SD, n = 3; ns: not significant). Note that the minor HBeAg ELISA signals in C-transfection samples were due to cross reaction with HBcAg.
(TIF)

S8 Fig Endogenous Alix is required for naked capsid secretion. HepG2 cells were transfected with siRNA si-con or si-Alix for 24 h, followed by pHBc transfection for 3 additional days. The expression of transfected HBc and endogenous Alix was analyzed by Western blot, β-actin served as a loading control. Intracellular and extracellular capsids were detected by particle gel immunoblot.
(TIF)

S9 Fig Alix ubiquitination assay in 293T cells. 293T cells were transfected with HA-Alix and control vector or His-Ub for 3 days. The expression of transfected HA-Alix and His-Ub was analyzed by Western blot. HA-Alix immunoprecipitation was performed and the co-immunoprecipitated HA-Alix and ubiquitinated Alix (Ub-Alix) were detected by Western blot. Antibody heavy chain (Ab HC) was labeled.
(TIF)

S10 Fig Screening of NEDD4 E3 ubiquitin ligases for regulators of naked capsid secretion. HepG2 cells were transfected with pHBc and plasmid expressing each NEDD4 E3 ubiquitin ligase family member for 3 days. The expression of each E3 ligase was confirmed by detecting the YFP tag signal under a fluorescence microscope. Intracellular and extracellular HBV capsids were detected by particle gel immunoblot.
(TIF)

S11 Fig AIP4 promotes the release of HBV DNA- and pgRNA-containing naked capsids in 293T cells. (A) Overexpression of AIP4 promotes the egress of HBV DNA-containing naked capsid. 293T cells were co-transfected with envelope-null pTREHBVDES and pTet-off together with control vector or Myc-AIP4 for 5 days. The intracellular HBV total RNA, HBc, Myc-AIP4, cytoplasmic HBV capsid and DNA content, and core DNA intermediates were analyzed by Northern bot (upper panel), Western blot (upper middle panels), capsid gel (middle panels), and Southern blot (lower middle panel), respectively. Extracellular naked capsids and viral DNA content were detected by particle gel immunoblot with HBcAb and hybridization with (-) strand-specific HBV riboprobe, respectively. (B) AIP4 promotes the egress of HBV pgRNA-containing naked capsids. 293T cells were co-transfected with priming-defective and envelope-null pCMVHBVY63DΔS and control vector or Myc-AIP4 for 3 days. The intracellular HBV total RNA, HBc, Myc-AIP4, cytoplasmic HBV capsid and core DNA intermediates, and extracellular naked capsid were analyzed as above described. The intracellular and extracellular capsid-associated HBV RNA were detected by particle gel hybridization using a (+) strand-specific full-length HBV riboprobe.
(TIF)

S12 Fig Intracellular colocalization of AIP4 and Alix. HepG2 **c**ells were co-transfected with Myc-AIP4 and HA-Alix for 3 days, followed by immunofluorescence confocal microscopy analysis of Myc-AIP4 (stained in red) and HA-Alix (stained in green), their colocalization was shown as bright yellow signals. Cell nuclei were stained by DAPI (blue). Three representative microscopic fields are shown. Image J colocalization finder was used for analyzing localization of Myc-AIP4 and HA-Alix.
(TIF)

S1 Data Source data for quantitative analyses in this paper. (XLSX)
