# Supplementary figures and images for: NEDD4 family ubiquitin ligase AIP4 interacts with Alix to enable HBV naked capsid egress in an Alix ubiquitination-independent manner
Source: PLoS Pathog. 2024 Sep 11;20(9):e1012485. doi: 10.1371/journal.ppat.1012485 (PMC11389946; doi:10.1371/journal.ppat.1012485)

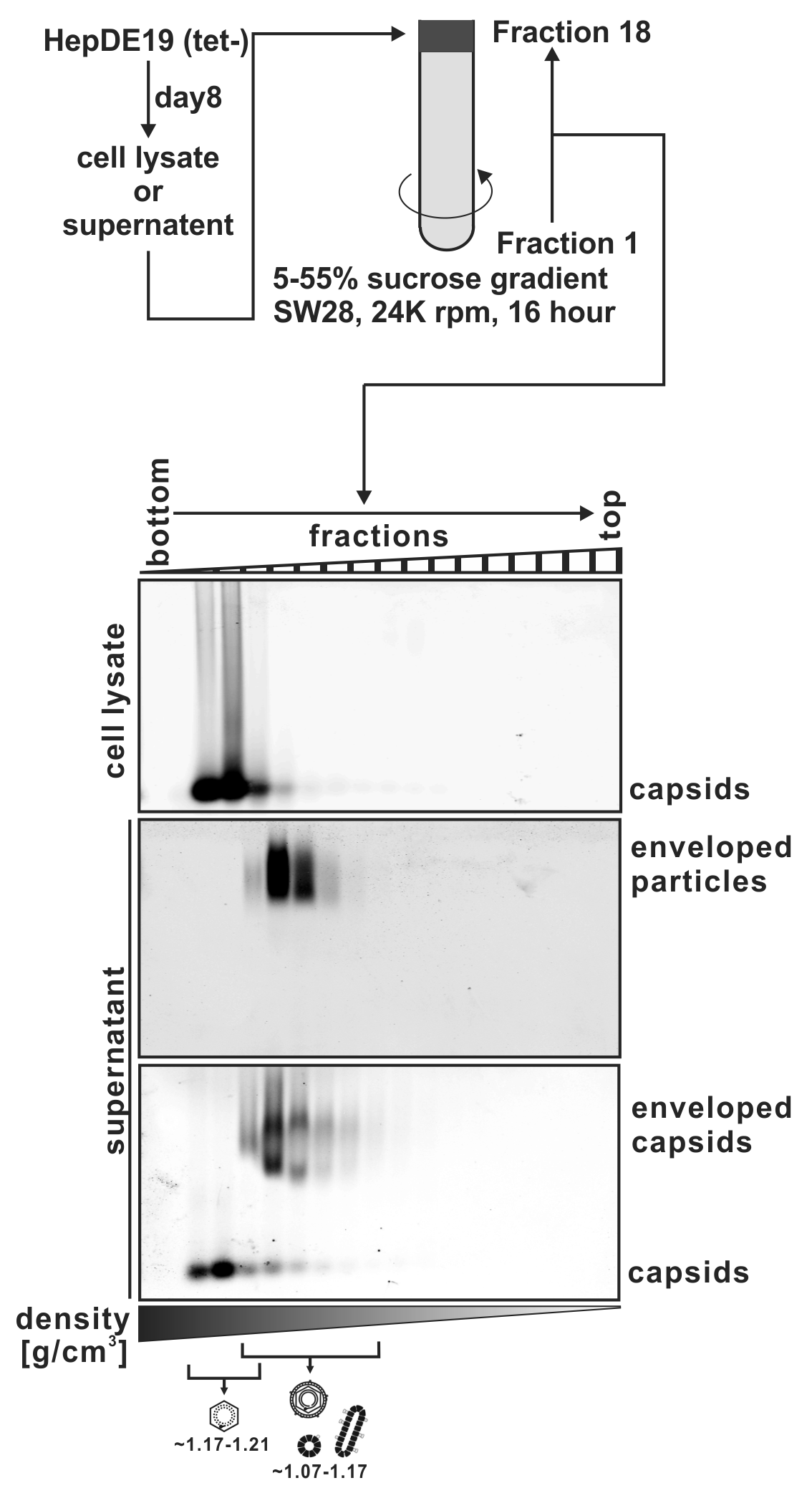

Supplement: S1 Fig — The cytoplasmic lysate and supernatant of induced HepDE19 cells (tet-, day 8) were ultracentrifuged in the 5–55% sucrose gradient, followed by the collection of fractions from the bottom of the gradient. Each fraction was analyzed for sucrose density using a refractometer and subjected to particle gel immunoblot assay to detect cytoplasmic capsids, extracellular enveloped particles, and naked capsids. Note that the intracellular and extracellular capsids exhibit a similar density range. (TIF) [file ppat.1012485.s001.tif]

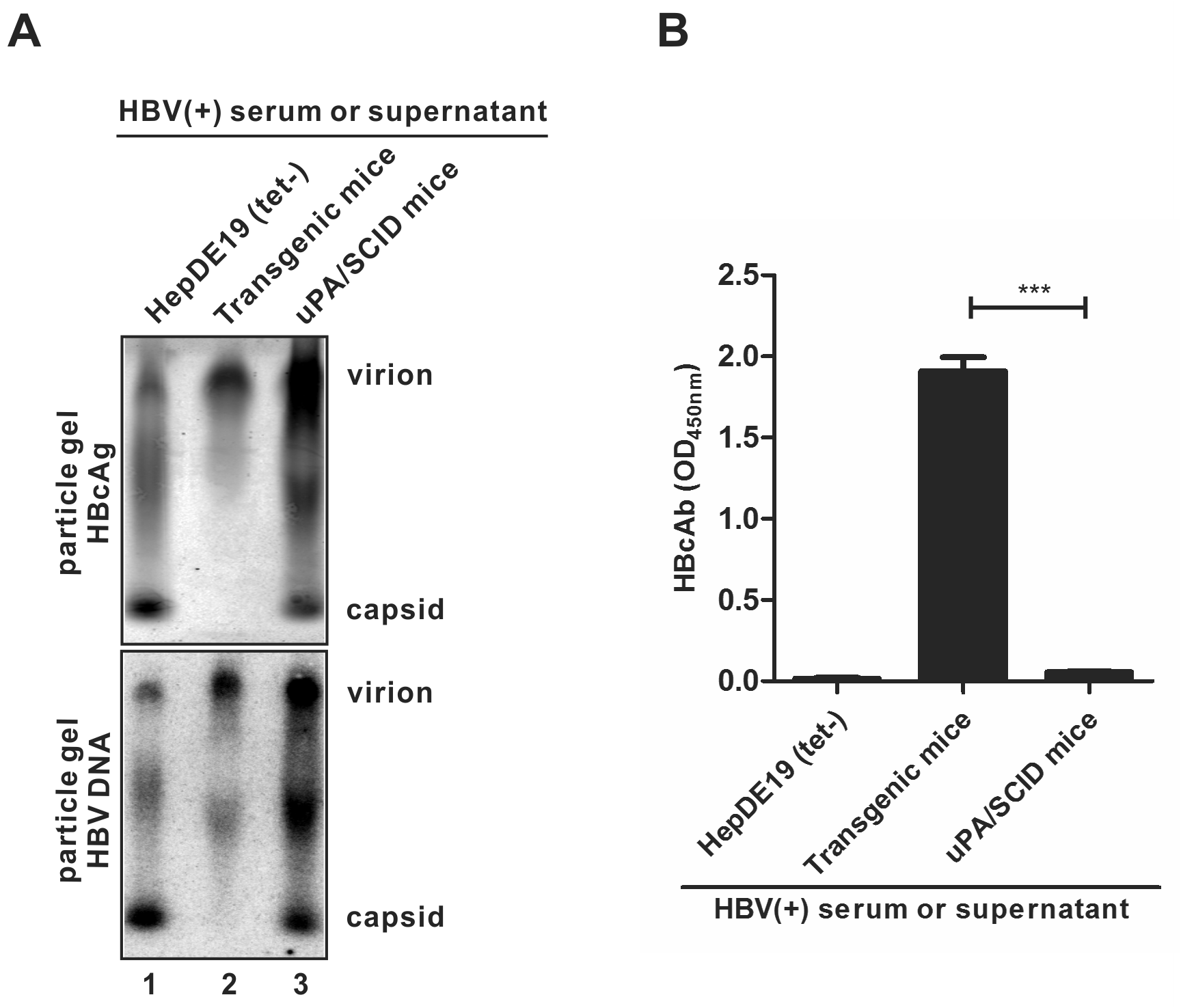

Supplement: S2 Fig — The induced HepDE19 cell culture fluid and indicated animal sera were subjected to (A) particle gel assay to detect HBV capsids and capsid-associated DNA by immunoblot and hybridization, respectively; and (B) HBcAb ELISA (mean ± SD, n = 3; ***p<0.001). (TIF) [file ppat.1012485.s002.tif]

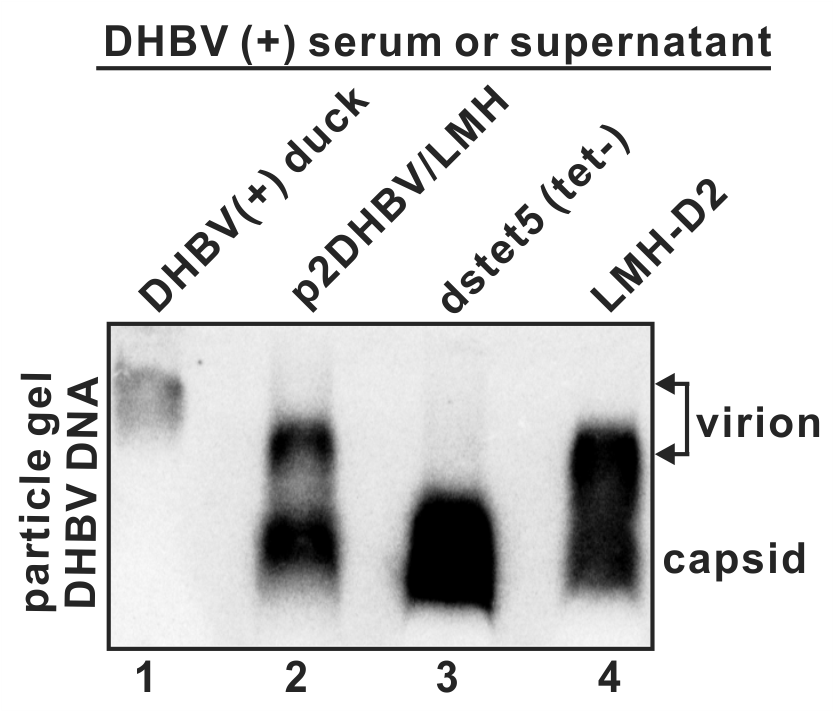

Supplement: S3 Fig — DHBV-positive duck serum and supernatant of DHBV-transfected LMH cells and DHBV stable cell lines (dstet5 cells and LMH-D2 cells) were subjected to particle gel assay, virion, and naked capsid were revealed by hybridizing their viral DNA content with (-) strand-specific DHBV riboprobe. (TIF) [file ppat.1012485.s003.tif]

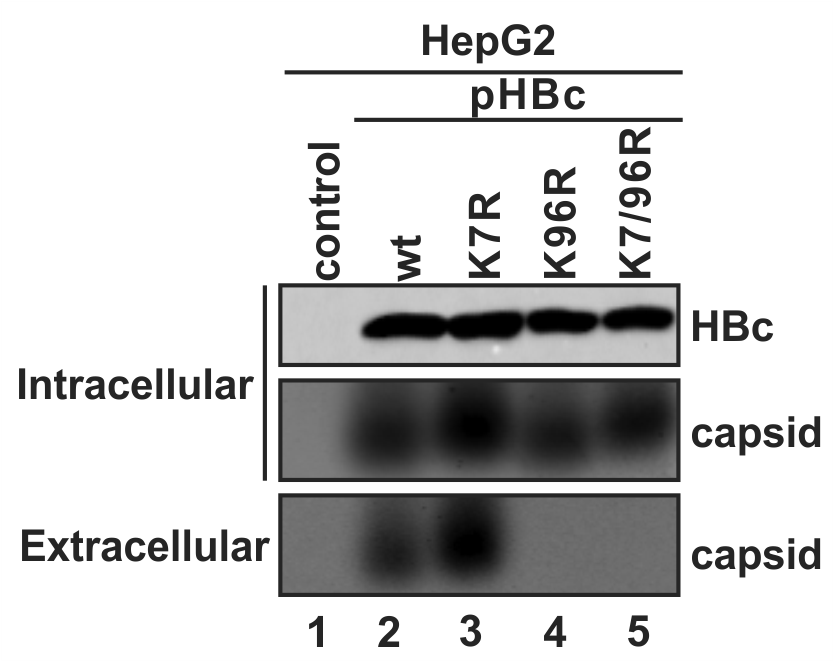

Supplement: S4 Fig — Plasmid expressing wt HBc or each mutant (K7R, K96R, K7/96R) was transfected into HepG2 cells for 3 days, followed by Western blot analysis of wt and mutant HBc proteins, and particle gel immunoblot assay of intracellular and extracellular capsids. (TIF) [file ppat.1012485.s004.tif]

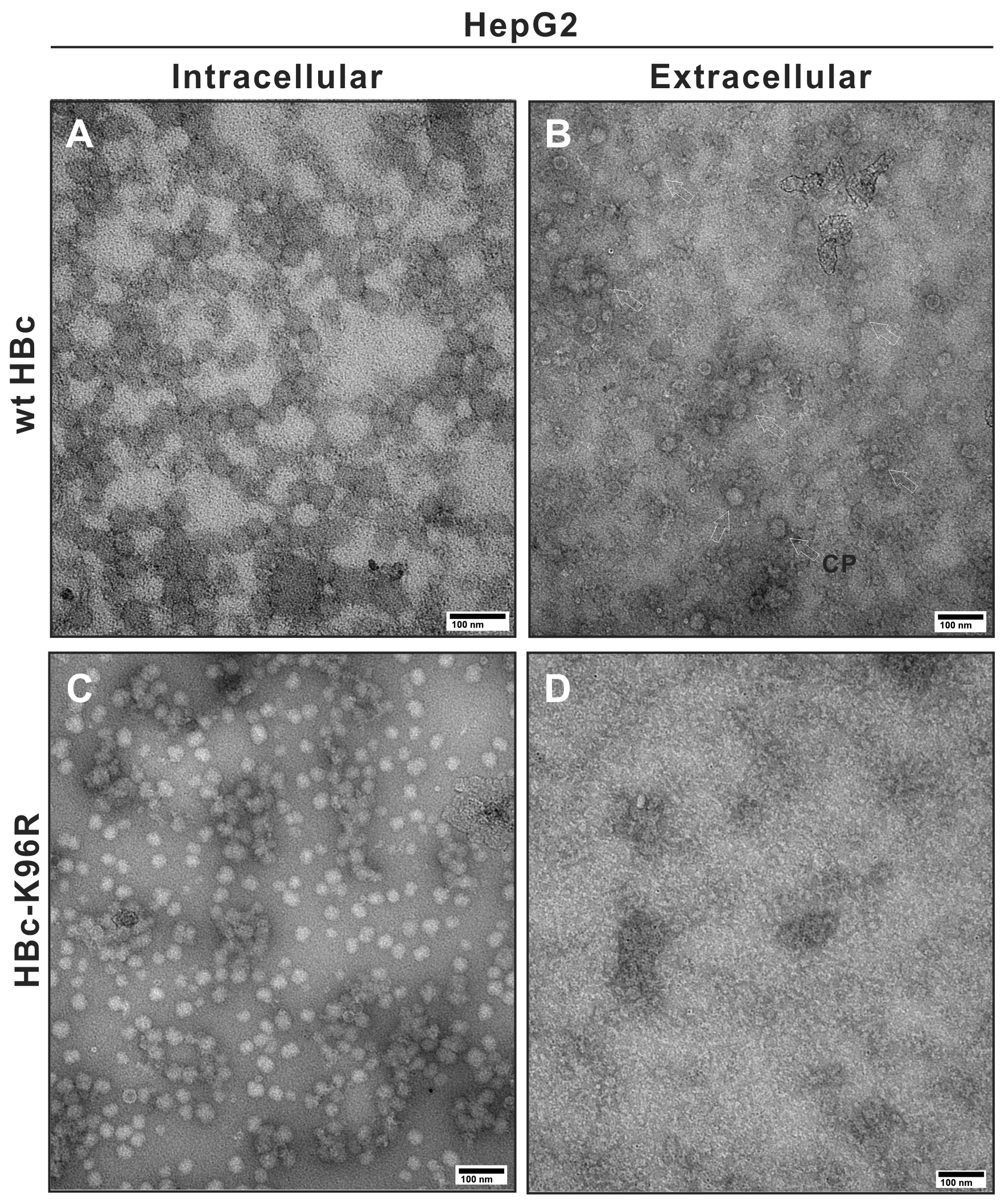

Supplement: S5 Fig — HepG2 cells were transfected with wt HBc or HBc-K96R mutant for 5 days. The intracellular (A, C) and extracellular (B, D) capsid particles were prepared as described in Materials and Methods and subjected to TEM visualization. Scale bar: 100 nm. (TIF) [file ppat.1012485.s005.tif]

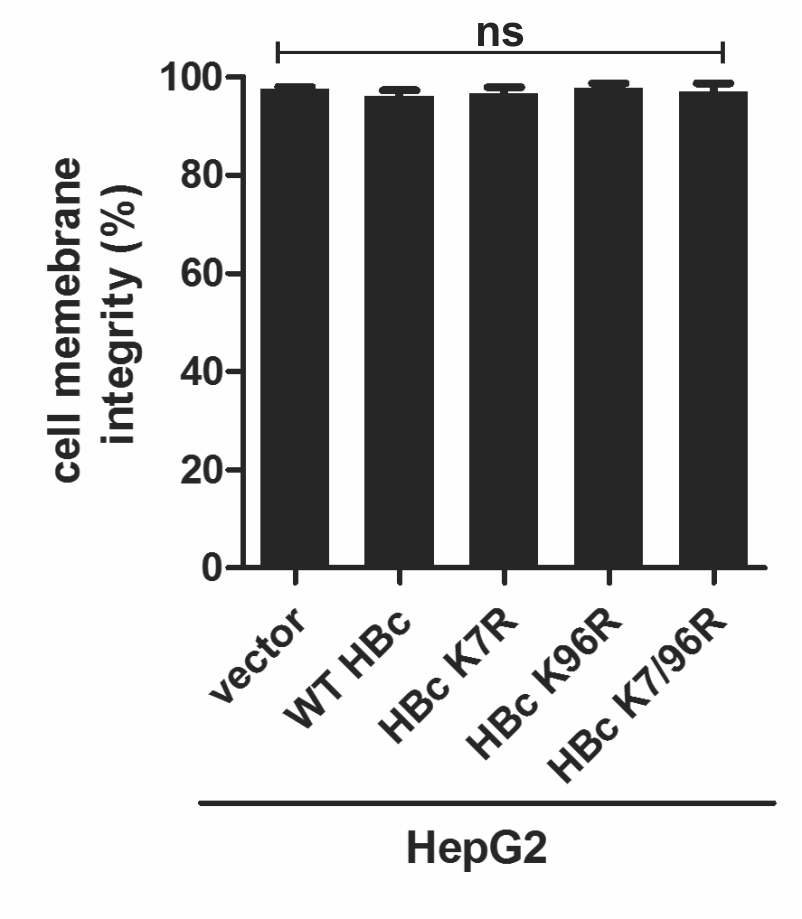

Supplement: S6 Fig — HepG2 cells were transfected with the control vector or indicated wt or mutant HBc expression vector for 5 days, the release of LDH was measured by CytoTox-ONE Homogeneous Membrane Integrity Assay, and the relative cell viability values were calculated according to the manufacturer’s manual and plotted as the percentage of the value from control samples (mean ± SD, n = 3; ns: not significant). (TIF) [file ppat.1012485.s006.tif]

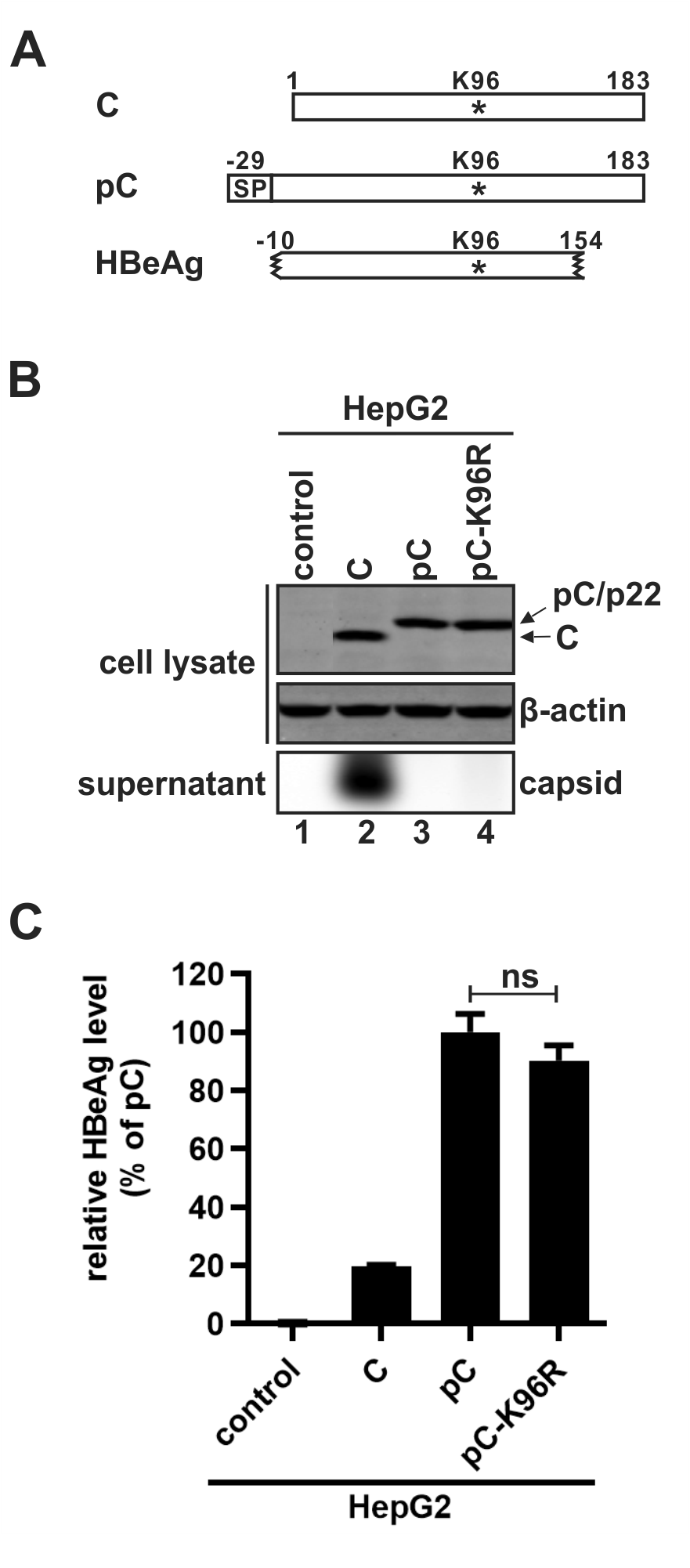

Supplement: S7 Fig — (A) Schematic representation of HBV C (HBc), pC, and HBeAg proteins. The aa positions are labeled with numbers, the first aa of C is set as position 1 and the position of K96 in each protein is marked with an asterisk. The pC protein possesses the same aa sequence with HBc plus the N-terminal 29 aa extension including a 19 aa signal peptide. The signal peptide sequence of pC is co-translationally cleaved, giving rise to a p22 intermediate, which is further processed by Furin peptidase in Golgi to remove the C-terminal domain and secreted as HBeAg. (B) HepG2 cells were transfected with plasmid expressing wt C, or wt pC, or pC-K96R mutant for 3 days. The expression of C, pC (p22), and pC-K96R was analyzed by Western blot, β-actin served as a loading control, intracellular capsids were revealed by capsid gel immunoblot. (C) HBeAg in the supernatant of above transfected cells was detected by ELISA and plotted as percentage of the pC-transfection positive control (mean ± SD, n = 3; ns: not significant). Note that the minor HBeAg ELISA signals in C-transfection samples were due to cross reaction with HBcAg. (TIF) [file ppat.1012485.s007.tif]

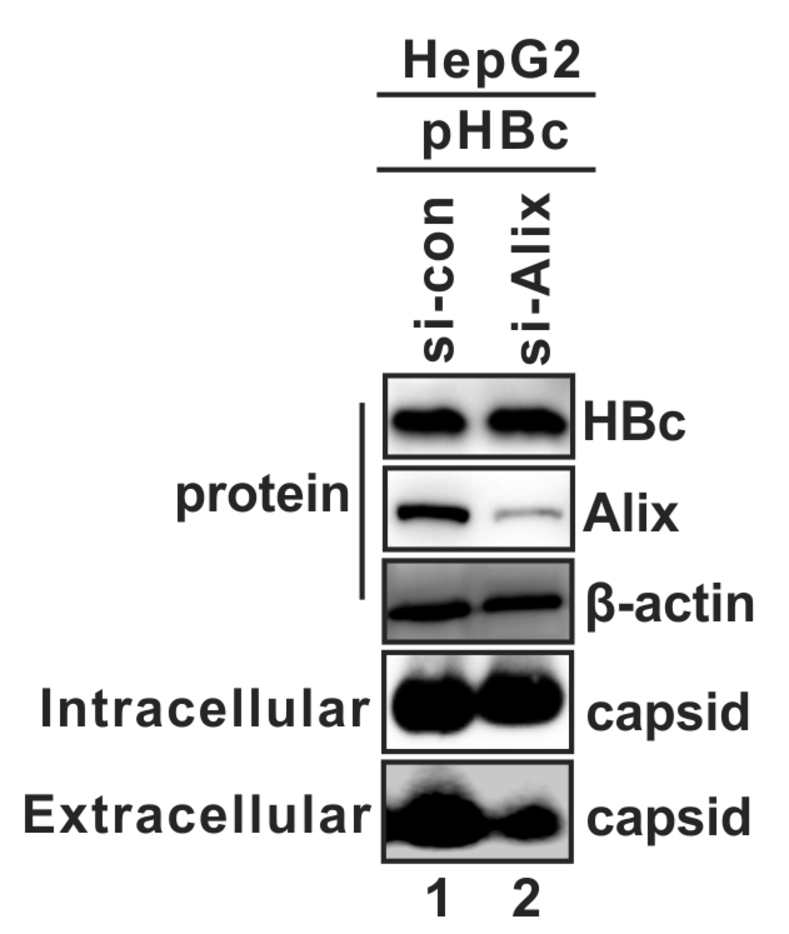

Supplement: S8 Fig — HepG2 cells were transfected with siRNA si-con or si-Alix for 24 h, followed by pHBc transfection for 3 additional days. The expression of transfected HBc and endogenous Alix was analyzed by Western blot, β-actin served as a loading control. Intracellular and extracellular capsids were detected by particle gel immunoblot. (TIF) [file ppat.1012485.s008.tif]

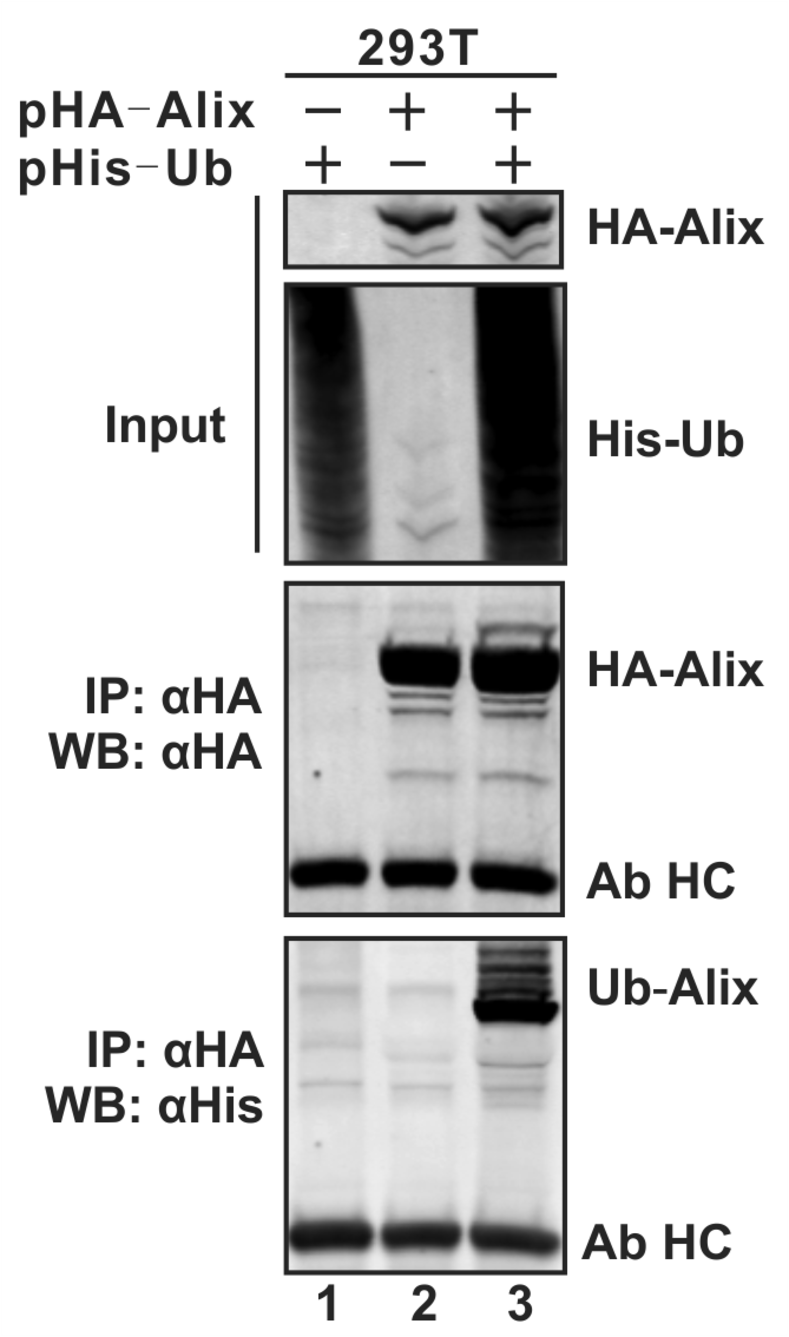

Supplement: S9 Fig — 293T cells were transfected with HA-Alix and control vector or His-Ub for 3 days. The expression of transfected HA-Alix and His-Ub was analyzed by Western blot. HA-Alix immunoprecipitation was performed and the co-immunoprecipitated HA-Alix and ubiquitinated Alix (Ub-Alix) were detected by Western blot. Antibody heavy chain (Ab HC) was labeled. (TIF) [file ppat.1012485.s009.tif]

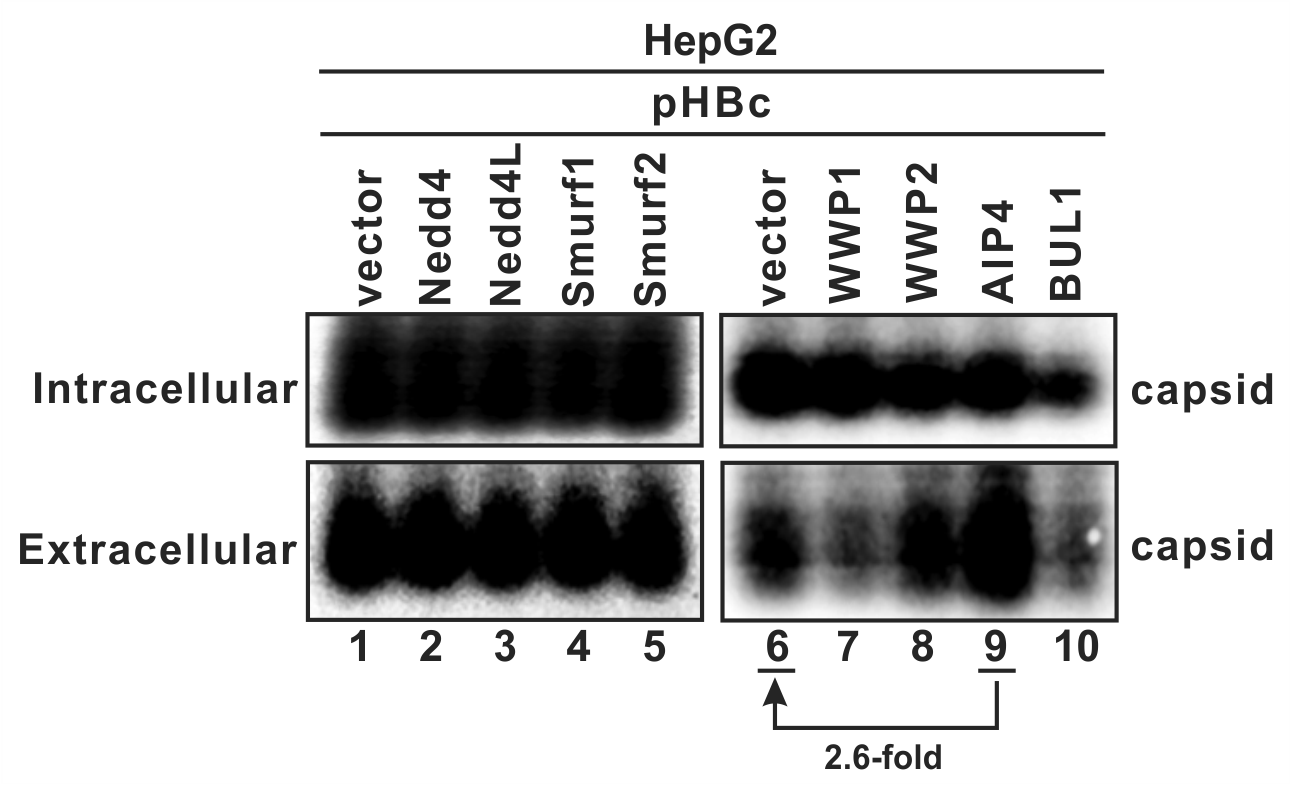

Supplement: S10 Fig — HepG2 cells were transfected with pHBc and plasmid expressing each NEDD4 E3 ubiquitin ligase family member for 3 days. The expression of each E3 ligase was confirmed by detecting the YFP tag signal under a fluorescence microscope. Intracellular and extracellular HBV capsids were detected by particle gel immunoblot. (TIF) [file ppat.1012485.s010.tif]

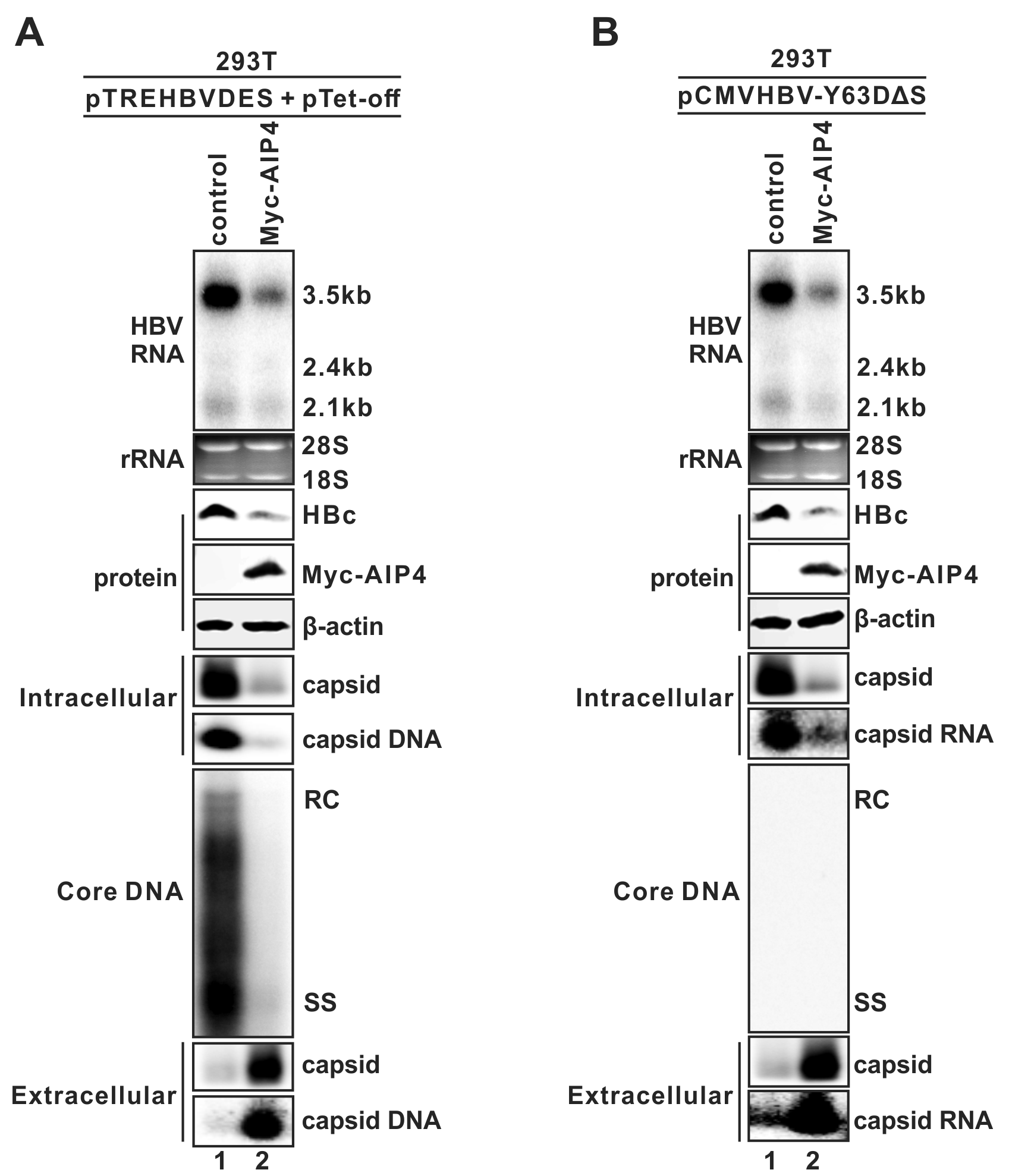

Supplement: S11 Fig — (A) Overexpression of AIP4 promotes the egress of HBV DNA-containing naked capsid. 293T cells were co-transfected with envelope-null pTREHBVDES and pTet-off together with control vector or Myc-AIP4 for 5 days. The intracellular HBV total RNA, HBc, Myc-AIP4, cytoplasmic HBV capsid and DNA content, and core DNA intermediates were analyzed by Northern bot (upper panel), Western blot (upper middle panels), capsid gel (middle panels), and Southern blot (lower middle panel), respectively. Extracellular naked capsids and viral DNA content were detected by particle gel immunoblot with HBcAb and hybridization with (-) strand-specific HBV riboprobe, respectively. (B) AIP4 promotes the egress of HBV pgRNA-containing naked capsids. 293T cells were co-transfected with priming-defective and envelope-null pCMVHBVY63DΔS and control vector or Myc-AIP4 for 3 days. The intracellular HBV total RNA, HBc, Myc-AIP4, cytoplasmic HBV capsid and core DNA intermediates, and extracellular naked capsid were analyzed as above described. The intracellular and extracellular capsid-associated HBV RNA were detected by particle gel hybridization using a (+) strand-specific full-length HBV riboprobe. (TIF) [file ppat.1012485.s011.tif]

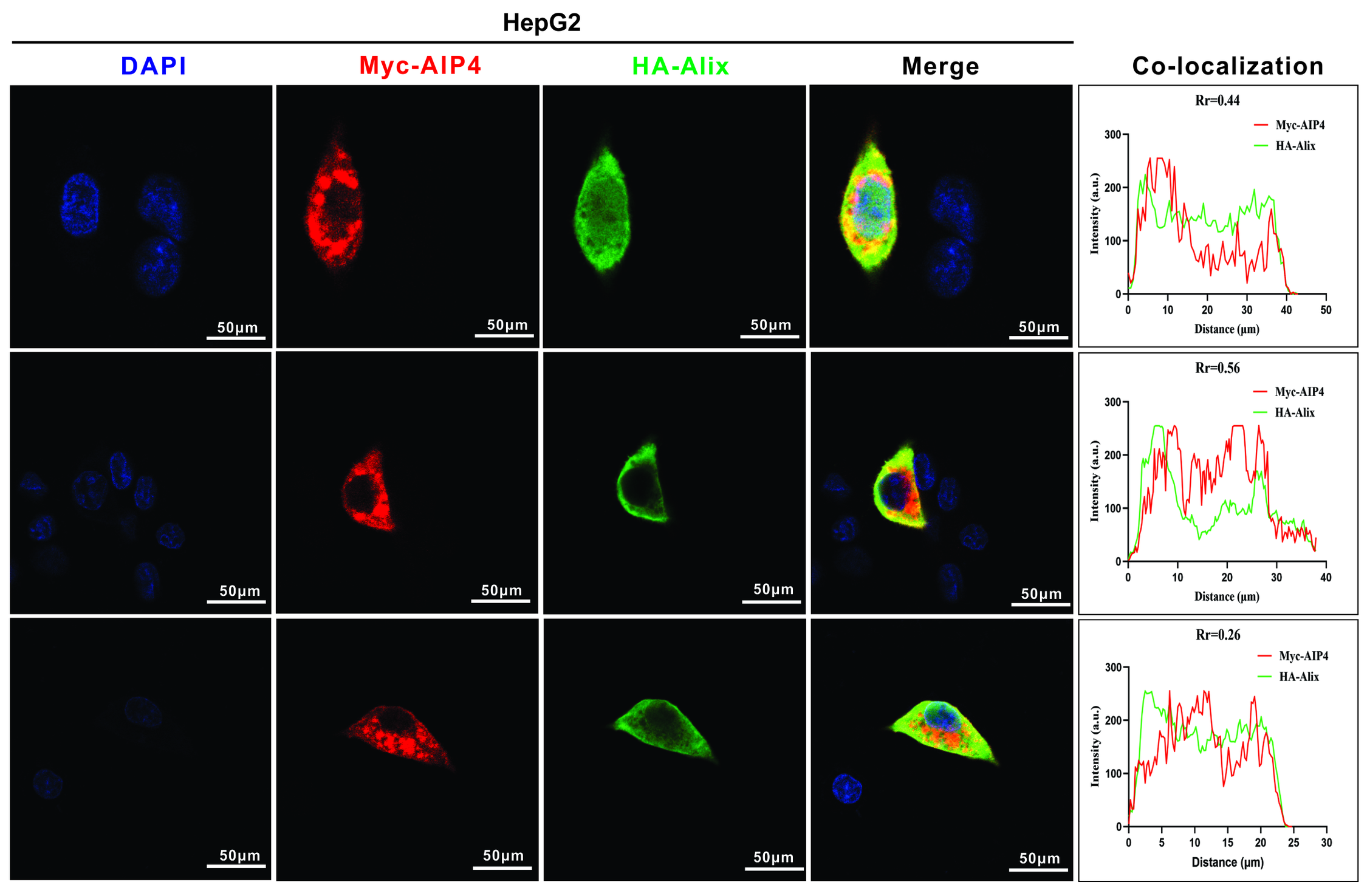

Supplement: S12 Fig — HepG2 cells were co-transfected with Myc-AIP4 and HA-Alix for 3 days, followed by immunofluorescence confocal microscopy analysis of Myc-AIP4 (stained in red) and HA-Alix (stained in green), their colocalization was shown as bright yellow signals. Cell nuclei were stained by DAPI (blue). Three representative microscopic fields are shown. Image J colocalization finder was used for analyzing localization of Myc-AIP4 and HA-Alix. (TIF) [file ppat.1012485.s012.tif]
